# Accumulation of pTreg cells is detrimental in late‐onset (aged) mouse model of multiple sclerosis

**DOI:** 10.1111/acel.13630

**Published:** 2022-05-26

**Authors:** Weikan Wang, Rachel Thomas, Jiyoung Oh, Dong‐Ming Su

**Affiliations:** ^1^ 12376 Department of Microbiology, Immunology, and Genetics University of North Texas Health Science Center Fort Worth Texas USA; ^2^ Alcon Research, LLC Fort Worth Texas 76134 USA; ^3^ Department of Pediatrics University of Texas Southwestern Medical Center Dallas Texas 75390 USA

**Keywords:** aging, autoimmunity, negative selection, regulatory T cell generation, thymic atrophy

## Abstract

Although typically associated with onset in young adults, multiple sclerosis (MS) also attacks the elderly, which is termed late‐onset MS. The disease can be recapitulated and studied in a mouse model, experimental autoimmune encephalomyelitis (EAE). The onset of induced EAE is delayed in aged mice, but disease severity is increased relative to young EAE mice. Given that CD4^+^FoxP3^+^ regulatory T (Treg) cells play an ameliorative role in MS/EAE severity, and the aged immune system accumulates peripheral Treg (pTreg) cells, failure of these cells to prevent or ameliorate EAE disease is enigmatic. When analyzing the distribution of Treg cells in EAE mice, the aged mice exhibited a higher proportion of polyclonal (pan‐) pTreg cells and a lower proportion of antigen‐specific pTreg cells in the periphery but lower proportions of both pan‐ and antigen‐specific Treg cells in the central nervous system (CNS). Furthermore, in the aged inflamed CNS, CNS‐Treg cells exhibited a higher plasticity, and T effector (CNS‐Teff) cells exhibited greater clonal expansion, disrupting the Treg/Teff balance. Transiently inhibiting FoxP3 or depleting pTreg cells partially corrected Treg distribution and restored the Treg/Teff balance in the aged inflamed CNS, thereby ameliorating the disease in the aged EAE mice. These results provide evidence and mechanism that accumulated aged pTreg cells play a detrimental role in neuronal inflammation of aged MS.

## Graphical abstract

Aged mice have accumulated peripheral Treg cells (pTregs) with severe EAE symptoms. Treg distribution is disrupted outside and inside the inflamed CNS, combined with increased CNS‐Treg plasticity. Transient inhibition of accumulated pTregs can redistribute Treg cells and mitigate the symptoms.

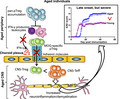

**Abbreviations**
AD: Alzheimer's disease
Ag: antigen
BBB: blood–brain barrier
CNS: central nervous system
CNS‐Teff: CNS‐infiltrated Teff cells
CNS‐Treg: CNS‐infiltrated Treg cells
CP: choroid plexus
DPI: day postimmunization
DT: diphtheria toxin
DTR: diphtheria toxin receptor
EAE: experimental autoimmune encephalomyelitis
GO: Gene Ontology
LN: lymph nodes
MFI: mean florescence intensity
MOG: myelin oligodendrocyte glycoprotein
MOG‐sp.: MOG‐specific
MS: multiple sclerosis
pan‐: polyclonal
PPMS: primary progressive MS
PRMS: progressive‐relapsing MS
PT: pertussis toxin
pTeff: peripheral Teff cell
pTreg or tTreg: peripheral Treg or thymic Treg
RRMS: relapsing‐remitting MS
sc‐RNA‐Seq: single‐cell RNA sequencing
SPMS: secondary progressive MS
Teff: T effector cell
Treg: regulatory T cell

## 1 INTRODUCTION

Neuronal inflammatory diseases commonly occur in the elderly. These diseases are tightly associated with aberrant immune cell function (Coder et al., 2016). Human multiple sclerosis (MS) is an autoimmune neuronal inflammatory disease, and its pathology is mainly associated with CD4^+^ T effector (Teff) cell‐mediated autoimmune demyelination (McFarland & Martin, 2007). Although it typically presents with onset in young adults, it either persists in a relapsing‐remitting (RRMS) manner throughout the life of the patients, or it develops in the elderly (termed: late‐onset MS; Bermel et al., 2010; Polliack et al., 2001; Sanai et al., 2016). MS in the elderly exhibits a severe progression mediated by the aged immune milieu (Bolton & Smith, 2017; Stern et al., 2010). To study human MS, the experimental autoimmune encephalomyelitis (EAE) mouse model has been widely used. However, most of these studies, either human MS or mouse model EAE, have focused on young subjects and have thus overlooked the age‐associated T cell immune conditions. Therefore, there is insufficient knowledge regarding how MS/EAE is impacted by aged T cells, particularly aged CD4^+^ Teff and aged CD4^+^FoxP3^+^ regulatory T (Treg) cells.

T cell–mediated self‐tolerance for controlling autoimmunity (Bluestone et al., 2015), including neuronal autoimmunity, is established and maintained through two primary mechanisms (Klein et al., 2019): depleting self‐reactive T cell clones via thymocyte negative selection (Klein et al., 2014; Palmer, 2003) and suppressing aberrant immune reaction in the periphery via Treg cells (Bluestone & Tang, 2005; Dominguez‐Villar & Hafler, 2018), which are mainly generated by the thymus (Hsieh et al., 2012). The thymus undergoes a progressive age‐related involution during aging, which perturbs negative selection, resulting in an increased output of self‐reactive Teff cells to the periphery. These self‐reactive Teff cells attack self‐tissues inducing inflammation, thereby enhancing senescent somatic cell secretions, which underlie chronic inflammation (termed senescence‐associated secretory phenotype, SASP; Callender et al., 2018; Coppe et al., 2008, 2010) in the elderly (termed inflammaging; Giunta, 2006). Meanwhile, the aged thymus exhibits relatively enhanced thymic Treg (tTreg) generation (Oh et al., 2017), which join the accumulated peripheral Treg (pTreg) pool in the aged periphery (Raynor et al., 2012). This accumulation of polyclonal (pan)‐pTreg cells is disadvantageous for anti‐infection (Lages et al., 2008) and anti‐tumor (Curiel, 2008; Liu et al., 2011; Takeuchi & Nishikawa, 2016) immunity, and vaccination efficacy in the elderly (Casares et al., 2010). In elderly patients with neurodegenerative Alzheimer's disease (AD) or Parkinson's disease, the frequency of peripheral Treg cells and their expression of FoxP3 are increased. However, they do not ameliorate these diseases but rather are correlated with increased disease severity (Rosenkranz et al., 2007).

Given that Treg cells generally play an ameliorative role in MS disease onset and severity (Buc, 2013; Furtado et al., 2001; Kleinewietfeld & Hafler, 2014; Randolph & Fathman, 2006) and the aged immune system exhibits accumulated pan‐pTreg cells, we asked why these cells do not ameliorate the severity of MS in elderly patients. Mounting evidence shows that patients over the age of 65 are more likely to have a severe progressive course of MS. Primary progressive (PPMS, disease without remissions) and secondary progressive (SPMS, irreversible damage, and disability) are severe and commonly seen in aged patients. Additionally, aged patients are less likely to have a mild course, such as RRMS (<40%), compared with their young counterparts of whom >80% having RRMS (Minden et al., 2004; Sanai et al., 2016). We proposed that the answer to this question is found in the dichotomous roles of Treg cells during central nervous system (CNS) inflammation within the aged immune system (Coder et al., 2016). We believe that Treg cells have either a detrimental or beneficial role, which is dependent on their location, either inside the CNS (termed CNS‐Treg) or outside the CNS (in the periphery). In addition, the imbalance between Treg and Teff cells (Thomas et al., 2020) and Treg functional plasticity (Kleinewietfeld & Hafler, 2013) within the aged CNS could determine disease severity.

To verify our hypothesis, we investigated how accumulated aged pTreg cells impact late‐onset MS using the EAE model in aged mice by examining Treg distribution inside and outside the CNS, Treg antigen specificity to myelin oligodendrocyte glycoprotein (MOG) and pan‐antigens, and Treg function‐associated molecular profiles during EAE in young and aged animals. We also transiently inhibited FoxP3 expression in accumulated pan‐pTreg cells in the aged mice and demonstrated a partial amelioration of the disease in the aged mice. Finally, we verified the proposed underlying mechanism by which accumulated pTreg cells residing at a CNS barrier membrane, the brain choroid plexus (CP), potentially impede the trafficking of immune cells into the inflamed CNS. This results in disruption of the balance between Treg and Teff cells, thereby inhibiting Treg suppression of Teff cell clonal expansion in the inflamed CNS. In addition, CNS‐infiltrated Treg cells also showed increased plasticity exhibited by the co‐expression of IFN‐γ and/or IL‐17A along with FoxP3, which results in a reduction of suppressive capacity. Together, our results provide novel evidence that accumulated and compromised aged Treg cells do not play an ameliorative role but are potentially detrimental during late‐onset (aged) neuronal autoimmune EAE disease course and severity.

## 2 RESULTS

### 2.1 The course of late‐onset EAE disease of aged mice exhibited distinct characteristics

Since T cell‐mediated autoimmune MS disease typically has an onset in young adults, studies on MS etiology, pathology, immunology, disease courses, etc., have been focused on young patients (20–40 years old) or have used an EAE model with young mice (2–3 months old). However, late‐onset (aged) MS in the elderly (diagnosed at the age of 60 or over) has been reported (Bermel et al., 2010; Polliack et al., 2001; Sanai et al., 2016). More information about late‐onset MS necessitates an aged mouse model. Herein, we studied late‐onset EAE with mice ≥18 months of age.

**FIGURE 1:**
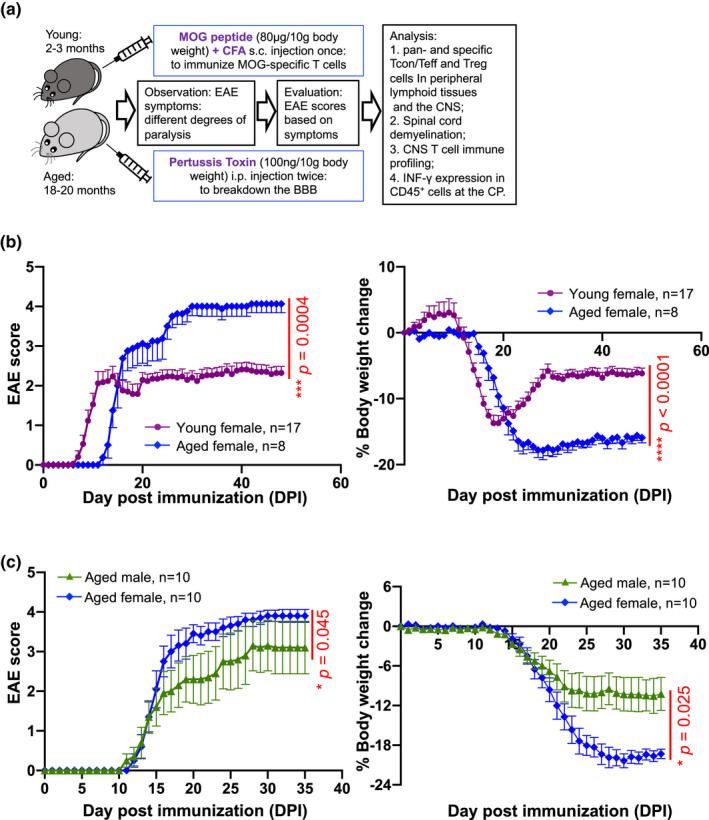
Characteristics of EAE disease in young versus aged mice. (a) Workflow for EAE induction in C57BL/6 mice with body weight‐dependent dosage and follow‐up analysis. (b) Characteristics of EAE pathological scores (left panel) and changes in body weights during disease (right panel) in young (cherry‐color line) versus aged (blue line) female mice. (c) The variability of EAE pathological scores (left panel) and changes in body weights during disease (right panel) in aged male and female mice. The *p*‐values of EAE scores were calculated by the Mann–Whitney *U* test, and body weight changes were calculated by two‐way repeated‐measures ANOVA with Geisser–Greenhouse correction, and a statistically significant difference was considered to be *p* < 0.05

To establish a late‐onset (aged) EAE mouse model, first, we immunized with a commonly used protocol (workflow in Figure S1a), in which the dosage is based on per mouse by injecting 200 µg MOG_35‐55_ peptide and 200 ng pertussis toxin (PT) to each young or aged C57BL/6 wild‐type (WT) female mice (Hooke Lab, etc.; Miller & Karpus, 2007). To our surprise, most aged mice showed a delayed onset (12‐day postimmunization, DPI) compared with the young (6‐DPI). Eight out of 13 (~2/3) of the aged mice showed no disease or very mild symptoms and never reached a debility score >3.0 by 48‐DPI (Figure S1b, blue line). This observation is contradictory to the increased symptom severity and progression in aged MS patients. As the body weight is a critical factor for the dosage administration of drugs, aged mice (30–40 g) have about 50% more to double the body weight of young mice (20–25 g; web links of C57BL/6 mouse age‐body weight information: See Figure S1 legend). We believed that the unexpected EAE progression/severity in the age mice might not be due to low susceptibilities to MOG_35‐55_ antigen but due to the insufficient MOG_35‐55_ peptide administration. Therefore, we adjusted the dosage of MOG_35‐55_ peptide to 80 µg/10 g body weight and PT to 100 ng/10 g body weight (Figure 1a, workflow). By comparing young and aged female mice, we determined that aged mice had a distinct EAE onset course. Aged mice indeed had a later EAE onset (~12‐DPI), which was about 6 days later than EAE onset in young mice (as early as at 6‐DPI); however, aged mice developed severer symptoms after onset (blue lines in Figure 1b, left panel: EAE scores; right panel: loss of body weight).

We next compared the courses of EAE disease in aged male (Figure 1c, green lines) and female mice (Figure 1c, blue lines) and found the two groups had similar EAE onset days, 11–12‐DPI. However, the male group had less severity and greater variation of disease symptoms, compared with the female mice. These characteristics are consistent with observations in human MS disease, in which women are the predominant population of MS patients, and also consistent with most published reports using female mice for EAE research (Reddy et al., 2005). Considering homogeneity of variance, we used female mice for the rest of our studies.

Taken together, these data provide novel evidence that pathological severity and course of EAE disease in young and old individuals are distinctively different. Therefore, using this aged EAE mouse model should provide insights into aged human MS onset, course, and severity.

### 2.2 Different distributions of Treg cells between young and aged mice during EAE disease

**FIGURE 2:**
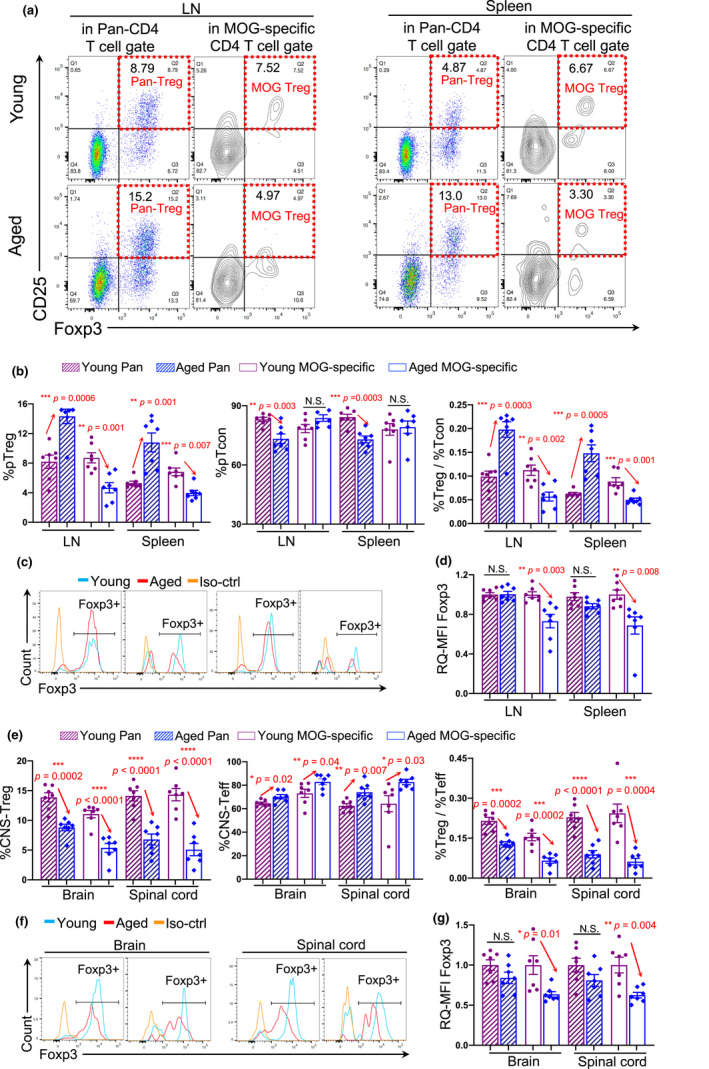
Imbalanced distributions of pan‐ and MOG‐specific Treg cells in the periphery and CNS of young and aged EAE mice. Mice were immunized as Figure 1a workflow. T cells from the LNs, spleen, brain, and spinal cord were analyzed. (a) Flow cytometry gating strategies show pan (polyclonal)‐ and MOG‐specific Treg cells in the LNs and spleens. MOG‐specific gate was determined by a dot plot of MHC‐II (I‐A^b^) MOG‐specific tetramer versus MHC‐II (I‐A^b^) control tetramer (Details in Figure S3). (b) Summarized results of the percentages of pan‐ (striped bars) and MOG‐specific (open bars) Treg (left panel) and Tcon (middle panel) cells, and ratios of Treg/Tcon cells (right panel) in the LNs and spleen between young (cherry) and aged (blue) EAE mice. (c) Flow cytometry gating strategies show FoxP3^+^ peaks from CD4^+^CD25^+^ gates of young and aged EAE mice. (d) Summarized results of the relative quantitative (RQ) mean fluorescence intensity (MFI) of FoxP3 expression in pan‐ (striped bars) and MOG‐specific (open bars) CD4^+^CD25^+^ population in the LNs and spleen between young (cherry) and aged (blue) EAE mice. (e) Summarized results of the percentages of pan‐ (striped bars) and MOG‐specific (open bars) Treg (left panel) and Teff (middle panel) cells, and ratios of Treg/Teff cells (right panel) in the CNS (the brain and spinal cord) between young (cherry) and aged (blue) EAE mice. (f) Flow cytometry gating strategies show FoxP3^+^ peaks from CD4^+^CD25^+^ gates of the CNS of young and aged EAE mice. (g) Summarized results of the RQ‐MFI of FoxP3 expression in pan‐ (striped bars) and MOG‐specific (open bars) CD4^+^CD25^+^ populations in the brain and spinal cord between young (cherry) and aged (blue) EAE mice. In panels (b), (d), (e) and (g), each symbol represents an individual animal sample. The *p*‐values between two groups were analyzed by unpaired the *Student's t*‐*test*, and a statistically significant difference was considered to be *p* < 0.05, “N.S.” stands for “not significant,” and error bars indicate mean ± SEM

Given that Treg cells play a vital protective role in the regulation of MS/EAE severity and progressive disease course (Buc, 2013; Furtado et al., 2001; Kleinewietfeld & Hafler, 2014; Randolph & Fathman, 2006), we compared the distributions of pan‐ and MOG‐specific (sp.) Treg cells, along with Teff (or termed conventional T, Tcon) cells, in the periphery (secondary lymphoid organs, Figure 2a–d, Figure S2a) and the CNS (brain and spinal cord, Figure 2e–g, Figure S2b,c) between young and aged mice during EAE disease (gating strategies are shown in Figure S3). We indeed found similarities and differences in the distributions between young and old, which hint at the possibility that Treg cells are involved in the characteristics of young and aged EAE disease.

In the periphery, either in the lymph node (LN) or spleen, the percentage (%) of pan‐pTreg cells in aged EAE mice was increased (Figure 2a,b stripped bars in left and right panels). This is consistent with published reports regarding the accumulation of pTreg cells in aged individuals (Raynor et al., 2012). However, % of MOG‐sp. pTreg cells in aged EAE mice was decreased (Figure 2a,b opened bars in left and right panels). The expression of FoxP3, which is tightly associated with Treg suppressive function, was decreased in MOG‐sp. pTreg cells of aged EAE mice (Figure 2c,d). In addition, % of pan‐Tcon cells was decreased (Figure 2b, striped bars in middle panel), but % of MOG‐sp. Tcon cells was not changed in aged EAE mice (Figure 2b, open bars in middle panel). The results suggest that although pan‐pTreg cells are increased, MOG‐sp. pTreg cells are reduced. This, coupled with unchanged MOG‐sp. pTcon cells in the aged periphery, results in an imbalance of MOG‐specific immunopathogenic and immunoregulatory T cells during aged EAE onset.

In the CNS, including the brain and spinal cord, the % of both pan‐CNS‐Treg and MOG‐sp. CNS‐Treg cells were decreased in aged EAE mice (Figure 2e left and right panels, Figure S2b). The results suggest an impaired pTreg trafficking into the aged, inflamed CNS. The expression of FoxP3 in MOG‐sp. CNS‐Treg cells within the aged, inflamed CNS was decreased (Figure 2f,g, Figure S2c), similar to those in the periphery (Figure 2c,d), suggesting that even if the MOG‐sp. Treg cells enter the aged CNS, they cannot fully execute their suppressive function. Particularly, both pan‐ and MOG‐sp. CNS‐Teff cells were increased in aged EAE mice (Figure 2e middle panel), different from those in the periphery (Figure 2b middle panel), suggesting that both CNS‐infiltrated pan‐ and MOG‐sp. Teff cells interact with the CNS tissues to participate in inflammation. However, both pan‐ and MOG‐sp. CNS‐Treg cells are less robust in controlling the inflammation in the CNS of aged EAE disease. Interestingly, such a discrepancy between the CNS‐Treg cells of young and aged mice was not observed at very early EAE onset without severe symptoms (Figure S4), suggesting that the imbalanced CNS‐Treg distribution in the aged mice is associated with EAE disease severity.

Together, these findings can help explain one of the reasons why aged mice have a late‐onset but more severe EAE pathology. The pTreg accumulation may delay MS/EAE onset at the early stage of the disease. However, once the disease onsets, the impaired trafficking of both pan‐ and MOG‐sp. Treg cells entering the CNS result in a lack of control of more severe progressive disease course. In other words, it is unlikely that the accumulation of pan‐pTreg cells in the periphery of aged mice can help mitigate EAE disease in aged individuals.

### 2.3 CNS‐infiltrated Treg cells in late‐onset EAE of aged mice exhibited dysfunctional molecular profiles

**FIGURE 3:**
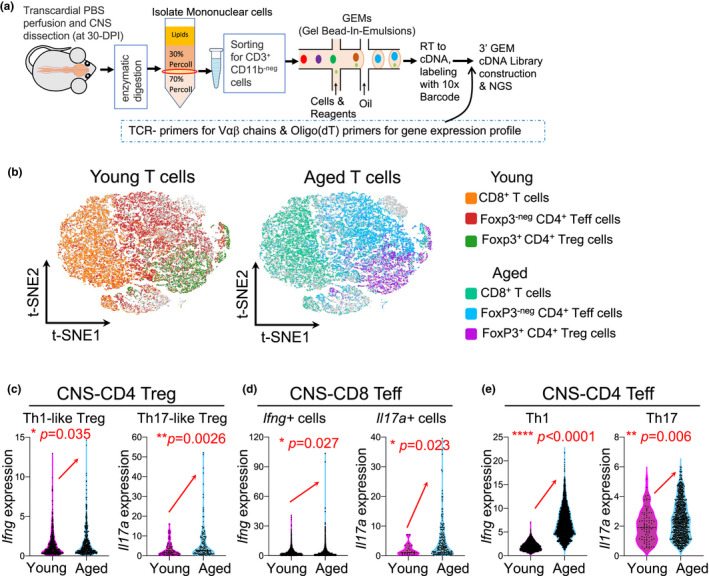
Function‐associated molecular profile analysis for CNS‐Treg plasticity and proinflammatory CNS‐CD8 T cells and CNS‐CD4 Teff cells of EAE mice. Mice were immunized per Figure 1a workflow. (a) Workflow of sc‐RNA‐seq for CNS‐infiltrated T cells. T cells in the CNS (the brain and spinal cord) were isolated via gradient centrifugation and sorted for CD3^+^CD11b^−neg^ cells by flow cytometry 30 days after immunization. Then, single cells were captured and emulsified with specific gel beads for reverse transcription and construction of cDNA library for high‐throughput sequencing. (b) The pattern of young (left t‐SNE plot) and aged (right t‐SNE plot) T cells from the CNS of three young and three aged EAE mice. (c–e) Normalized expressions of *Ifng* (left panel) and *Il17a* (right panel) genes in single Th1‐ and Th17‐like Treg (CD4^+^FoxP3^+^) cells (c), CD8^+^ T cells (d), and CD4^+^ Teff (CD4^+^FoxP3^−neg^) cells (e) of the young (cherry) and aged (blue) EAE CNS. Data in panels were analyzed by the unpaired *Student's t*‐*test*, and a statistically significant difference was considered to be *p* < 0.05. Each symbol represents a single cell

Treg cells can possess relatively unstable features (Rubtsov et al., 2010; Sawant & Vignali, 2014), including down‐regulation of FoxP3 expression (Bailey‐Bucktrout et al., 2013; Dominguez‐Villar & Hafler, 2018) as confirmed in our late‐onset (aged) EAE mouse model (Figure 2d,g), and the co‐expression of *Ifng* or *Il17a* toward Th1‐like or Th17‐like plastic conversion (Kitz & Dominguez‐Villar, 2017; McClymont et al., 2011) upon autoimmune stimulation (Bailey‐Bucktrout et al., 2013; Dominguez‐Villar & Hafler, 2018). This leads to functional plasticity, resulting in increased pathology and reduced suppressive capacity (Dominguez‐Villar & Hafler, 2018; Kitz & Dominguez‐Villar, 2017). We believe that this unstable phenotype is likely more prominent in the aged microenvironment. To evaluate these function‐related molecular profiles, we analyzed the expression of *Ifng* and *Il17a* genes in CNS‐infiltrated Treg cells of EAE mice at the single‐cell level (Figure 3a, workflow). We compared CD4^+^FoxP3^+^ CNS‐Treg cells in the young and aged inflamed CNS (Figure 3b) during EAE disease and found that the expression of *Ifng* and *Il17a* was indeed increased in the CNS‐infiltrated aged Treg cells (Figure 3c). In addition, we noticed that activated or pathogenic CNS‐CD8^+^ T cells, indicated by the expression of *Ifng* and *Il17a*, which potentially lead to autoimmune encephalomyelitis (Huber et al., 2013; Saxena et al., 2012), showed increased *Ifng* and *Il17a* expressions in the aged CNS with EAE disease (Figure 3d). In addition, in the typical EAE pathogenic CD4^+^ T cell subsets Th1 and Th17 cells, the expressions of *Ifng* and *Il17a* were increased in the aged, inflamed CNS (Figure3e). Gene Ontology (GO) enrichment analysis of CNS‐infiltrated CD4^+^ T cells in aged versus young EAE mice showed that T cell activation and proliferation pathways have enriched up‐regulated gene expression in aged CNS‐Teff cells (Figure S5a) but not in aged CNS‐Treg cells (Figure S5b). The results imply that aged Treg cells, which have infiltrated into the CNS after EAE onset, could have reduced capacity to suppress CNS‐Teff cell‐induced neuronal inflammation.

### 2.4 Clonal expansion of CNS‐infiltrated Teff cells in late‐onset EAE in aged mice was increased

**FIGURE 4:**
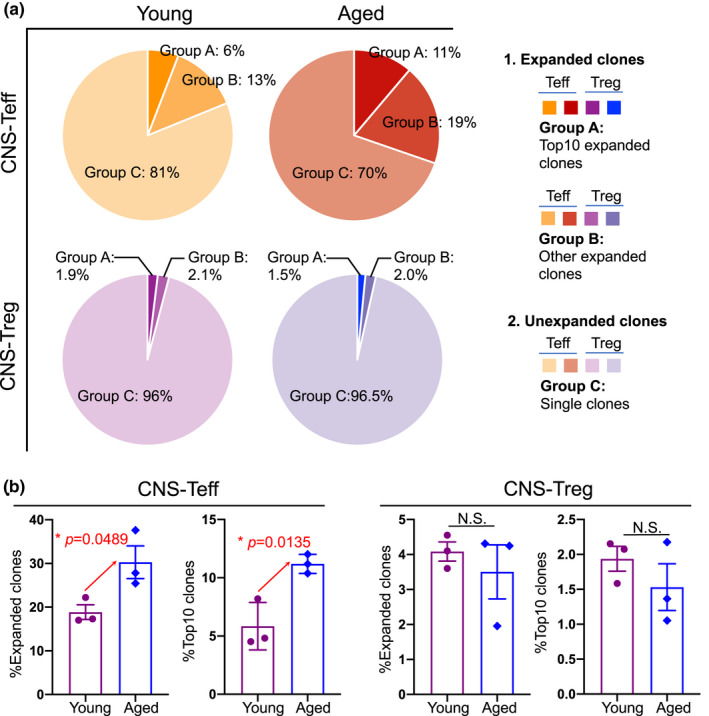
Clonal expansion assay in CNS‐infiltrated CD4^+^ T populations of young and aged EAE mice. Based on TCRαβ sequence similarity from the sc‐RNA‐Seq immune profile analysis, CNS‐infiltrated Teff and Treg cells were divided into expanded T clones (more than one copy of each TCR sequence) and unexpanded T clones (unique TCR sequences, with clone size = 1). In the expanded T clones, we further divided them into Group‐A: top 10 clones (10 most expanded TCR sequences) and Group‐B: other expanded clones (all other TCR sequences with ≥2 copies). (a) Pie charts show expanded clones (Groups‐A and ‐B) and unexpanded clones (Group‐C) of CNS‐Teff (top two pies) and CNS‐Treg (bottom two pies) cells in total CNS‐CD4^+^ T cells from young (left two pies) and aged (right two pies) EAE mice. (b) Summarized results of the frequencies of all expanded clones of CNS‐Teff (left panel) and CNS‐Treg (right panel) cells in total CNS‐CD4^+^ T cells from young (cherry open bars) and aged (blue open bars) EAE mice. Each symbol represents one mouse. Data were analyzed by the unpaired *Student's t*‐*test*, and a statistically significant difference was considered to be *p* < 0.05

To assess whether CNS‐infiltrated aged Treg cells have reduced suppressive capacity, an assessment of CNS‐Teff cell clonal expansion in the EAE CNS can shed some light. We therefore analyzed clonal expansion in the CNS‐infiltrated Teff cell pool along with the Treg cell pool based on TCR sequence similarity via TCRαβ immune profile analysis with single‐cell RNA sequencing (sc‐RNA‐Seq; Figure 4). The capacity of CNS‐Treg cells can be reflected by their suppression of clonal expansion of CNS‐Teff cells, which are the predominant T cells inducing EAE CNS inflammation. The results showed that aged CNS‐Teff cells had a greater clonal expansion, compared with the young (Figure 4a top pies and Figure S6 left two columns), and the expanded CNS‐Teff clones (both total expanded clones and top 10 expanded clones) occupied a higher proportion of the CNS‐Teff pool (Figure 4b left panels) in the aged, inflamed CNS. However, although Treg cells could proliferate in vivo (Walker et al., 2003), aged CNS‐Treg clones showed a similar expansion as in the young (Figure 4a bottom pies, b right panels, and Figure S6 right two columns). Together, aged CNS‐Teff cells are more activated in the aged, inflamed CNS during EAE disease, which is potentially due to insufficient suppression by CNS‐Treg cells. The results also indicate that the expansion capacity of CNS‐infiltrated Treg cells in aged EAE mice is not reduced compared with their young counterparts.

### 2.5 Transient inhibition or depletion of pan‐pTreg cells in the aged mice mitigated EAE severity

**FIGURE 5:**
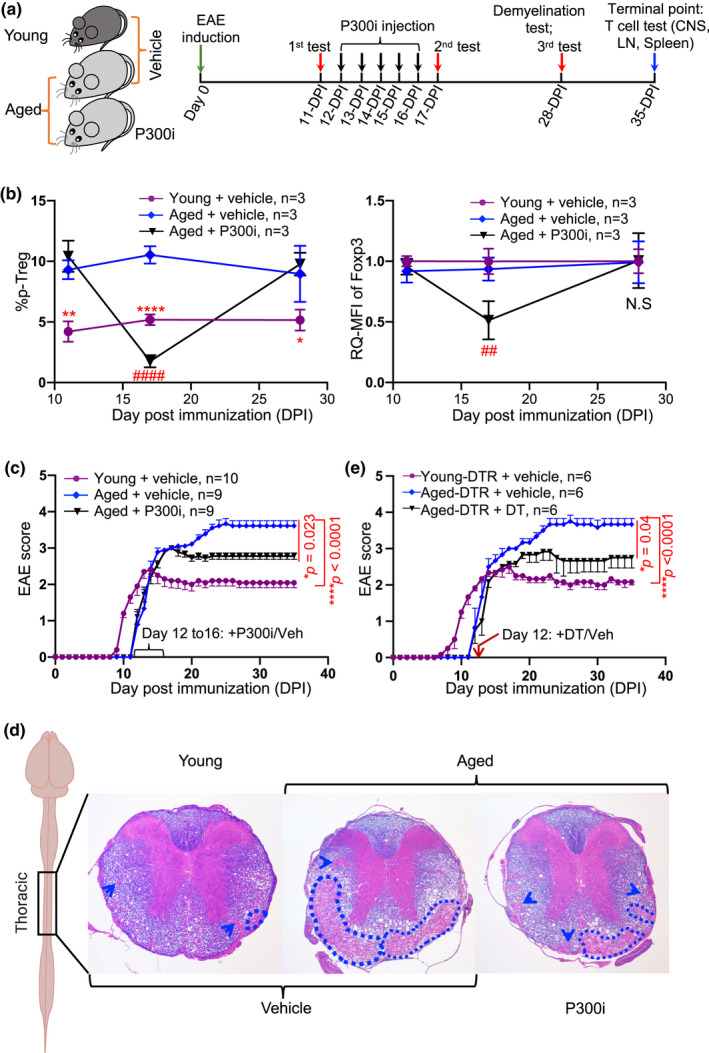
Alleviation of EAE severity after transient FoxP3 inhibition or depletion of the accumulated pTreg cells in aged EAE mice. (a) Workflow of EAE induction, transient inhibition of pTreg cells, blood collection time‐points (red arrowheads) for pTreg cell measurements, and demyelination assay. (b) Effects of transient inhibition of pTreg cells in aged mice. Higher pTreg percentage in aged mice than in young mice before the first injection of P300i or vehicle (11‐DPI, left panel), significantly reduced pTreg percentage and FoxP3 expression (MFI) one day after the last injection of P300i (17‐DPI, black triangles/line) in the aged EAE mice, and restored pTreg percentage in the aged EAE mice 12 days after the last P300i treatment (28‐DPI) to the control aged EAE mice (blue dots/line). All data are expressed as mean ± SEM and were analyzed by the one‐way ANOVA followed by the Dunnett's multiple post hoc test. *p < 0.05, **p < 0.01, and ****p < 0.0001, young + vehicle versus aged + vehicle; ^##^ *p* < 0.01 and ^####^ *p* < 0.0001, aged + vehicle versus aged + P300i. (c) Alleviation of the symptoms in late‐onset EAE of the 5x P300i‐treated aged group (black triangles/line) compared with the vehicle‐treated control aged group (blue dots/line). (d) Illustration of a mouse brain and spinal cord indicating the thoracic segment of the spinal cord for LFB‐eosin staining (rightmost illustration). Three representative spinal cord section images of LFB‐eosin staining, showing the alleviation of demyelination in the spinal cords of aged EAE mice treated with 5x P300i (right image), compared with their counterparts, the vehicle‐treated age‐matched control mice (middle image). The dotted outlines indicate areas of large foci of demyelination, and blue arrow heads show small foci of demyelination. This experiment was repeated three times in three mice of each group with essentially identical results. (e) Alleviation of the symptoms in late‐onset EAE of DT‐treated middle‐aged *FoxP3* ^DTR^ EAE mice. The “n” = animal numbers

Accumulation of pan‐pTreg cells in aged individuals (Raynor et al., 2012) was demonstrated to be harmful during neurodegenerative disease AD, since transient inhibition of FoxP3 expression in the accumulated pTreg cells attenuated AD pathology (Baruch et al., 2015). However, it is unknown whether this accumulation is detrimental or beneficial in late‐onset (aged) MS/EAE. Given that aged EAE disease occurs with accumulated pan‐pTreg cells in the periphery (Figure 2b), but less CNS‐Treg cells in the aged inflamed CNS (Figure 2e), we hypothesized that the cellular trafficking into the CNS may be impeded by the accumulation outside the CNS. Therefore, transient inhibition of FoxP3 expression in accumulated pTreg cells or transient depletion of accumulated pTreg cells in the aged mice could mitigate late‐onset EAE symptoms and pathology. Therefore, we tested two models. In the first model, we administrated 5‐doses of P300i to aged mice beginning on 12‐DPI (Figure 5a, workflow). P300i can impair Treg suppressive activities by inhibiting FoxP3 expression without affecting Teff cell responses (Liu et al., 2013) and has been used to transiently and partially suppress pan‐pTreg cells residing outside the CNS (Baruch et al., 2015). We evaluated the percentages of pTreg cells (Figure 5b left panel and Figure S7a) and expression level (via mean fluorescence intensity, MFI) of FoxP3 (Figure 5b right panel) in the peripheral blood at three time‐points (Figure 5a three red arrowheads indicate tests, i.e., before and after the P300i injections). The results show that the pan‐pTreg cells were decreased one day (11‐DPI) but returned to normal levels after 12 days (28‐DPI) after the last P300i injection (Figure 5b). This confirms that the inhibition was effective and transient, and pTreg cells had restored FoxP3 expression after the pharmaceutical inhibitory effects decayed over time.

Regarding the severity of EAE disease after transient inhibition of FoxP3 in accumulated pan‐pTreg cells in the aged mice, we evaluated EAE symptoms (disease scores and demyelination) and found that the severity was attenuated in the aged P300i‐treated group (Figure 5c black line and Figure 5d rightmost image). Although it did not restore the aged mice to the same level as the young group, it was significantly improved. The results provide evidence that the accumulation of aged pan‐pTreg cells in the periphery is not beneficial but rather detrimental to late‐onset EAE in aged mice.

To reconfirm the finding that impairing the accumulated pan‐pTreg in the aged mice can rescue from EAE symptoms, we tested the second model by introducing middle‐aged Foxp3‐DTR/EGFP mice (termed DTR mice) for the late‐onset EAE model. DTR mice have FoxP3^+^ Treg cells expressing diphtheria toxin receptor (DTR), and thus, Treg cells can be transiently depleted after administrating diphtheria toxin (DT; Kim et al., 2007). We intraperitoneally (i.p.) injected DT once to the aged DTR EAE mice with 50μg/kg body weight at 12‐DPI. We evaluated the percentages of pTreg cells in the peripheral blood one day before the injection (11‐DPI), one day after the injection (13‐DPI), and 16 days after the injection (28‐DPI). As expected, peripheral blood pTreg cells were completely depleted one day after the DT injection (Figure S7b, red box, and S7C) and restored to normal level 16 days (28‐DPI) after the injection (Figure S7b, blue box, and c). As a result, EAE symptoms were alleviated in the aged DTR mice with transient pTreg depletion (Figure 5e).

### 2.6 Transient inhibition or depletion of pan‐pTreg cells to open trafficking into the inflamed CNS was a potential mechanism of the EAE alleviation in aged mice

We wanted to elucidate the underlying mechanism by which late‐onset EAE alleviation occurred in aged mice via transient inhibition or depletion of pTreg cells. Based on a published report, accumulated pTreg cells, which are adherent to the CNS barrier sites, including the blood–brain barrier (BBB) and the brain CP, suppress IFN‐γ‐secreting cells, and potentially result in hampered trafficking of monocytes and antigen (Ag)‐specific Treg cells into the inflamed CNS (Raposo et al., 2014). Transient, rather than permanent, inhibition or depletion of the accumulated pTreg cells mitigated AD (Baruch et al., 2015) due to facilitated trafficking of anti‐inflammatory monocytes and Treg cells during CNS inflammation. We believe this is likely the case with late‐onset MS/EAE. Thus, we investigated the expression of IFN‐γ in CP‐adherent CD45^+^ hematopoietic cells (mononuclear cells, etc.) and the proportions of CNS‐infiltrated Treg and Teff cells with/without the transient inhibition in the aged mice.

**FIGURE 6:**
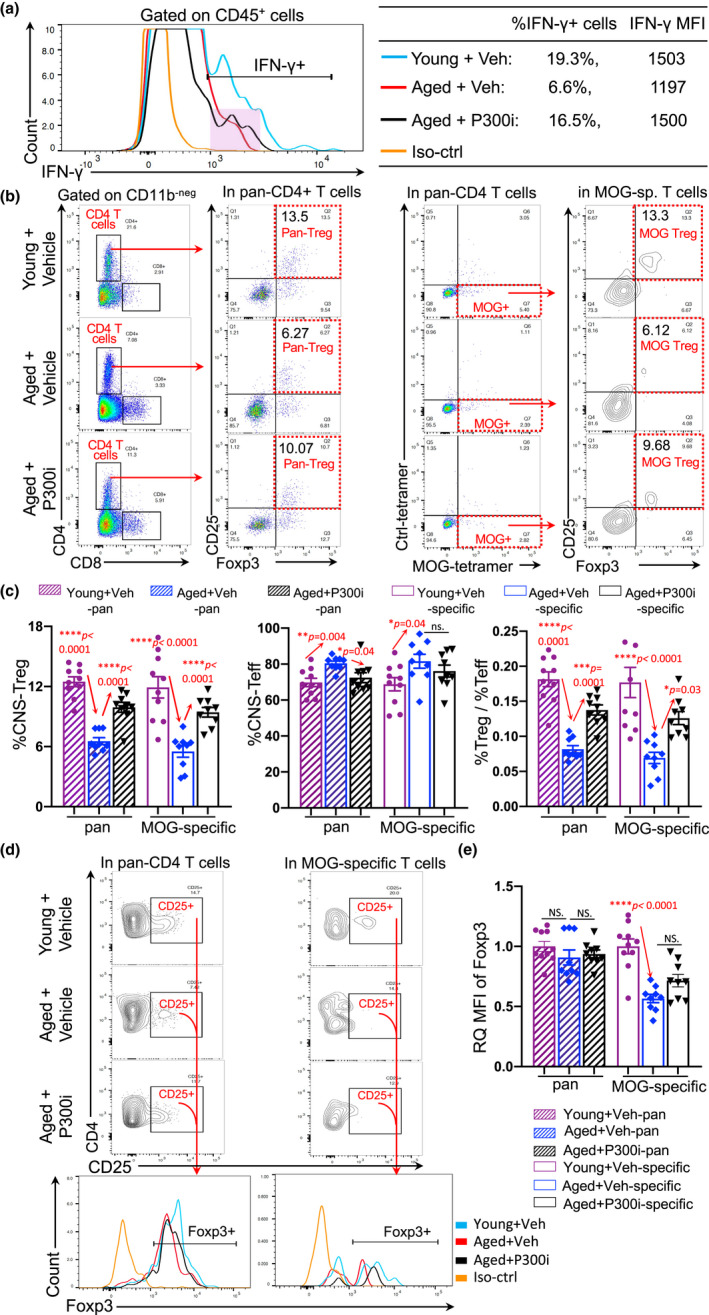
The alleviation of EAE in aged mice by transient inhibition of the accumulated pan‐pTreg cells was potentially due to restored immune cell trafficking. The workflow is shown in Figure 5a. (a) Expression of IFN‐γ^+^ in CP‐residing CD45^+^ cells were analyzed one day after the last P300i treatment (Figure 5a, the second test red arrow). A representative histogram shows IFN‐γ^+^ CD45^+^ cells residing at the CP isolated from the brains of young and aged EAE mice treated with P300i and vehicle, respectively. Percentages of IFN‐γ^+^ cells in CD45^+^ cells and MFI of IFN‐γ expression are listed in the table (right). (b) Flow cytometry gating strategies of pan‐Treg and MOG‐specific Treg cells in the CNS of young and aged EAE mice with P300i or vehicle treatment. (c) Summarized results of the percentages of pan‐ (striped bars) and MOG‐specific (opened bars) CNS‐Treg (left panel) and CNS‐Teff (middle panel) cells, and ratios of Treg/Teff cells (right panel) in the CNS (the brain and spinal cord) of young (cherry) and aged EAE mice, treated with vehicle (blue) or P300i (black). (d) Flow cytometry gating strategies show representative FoxP3^+^ gates (bottom panels) from CD4^+^CD25^+^ gates of the CNS of the three groups of EAE mice. (e) Summarized results of the RQ‐MFI of FoxP3 expression in pan‐ (striped bars) and MOG‐specific (open bars) CD4^+^CD25^+^ population in the CNS among young (cherry) and aged EAE mice treated with vehicle (blue) or P300i (black). In panels C and E, each symbol represents an individual animal sample. Data are expressed as mean ± SEM. The *p*‐values between three groups were analyzed by the one‐way ANOVA with a Dunnett's multiple post hoc test, and a statistically significant difference was considered to be *p* < 0.05, “NS.” stands for “not significant”

**FIGURE 7:**
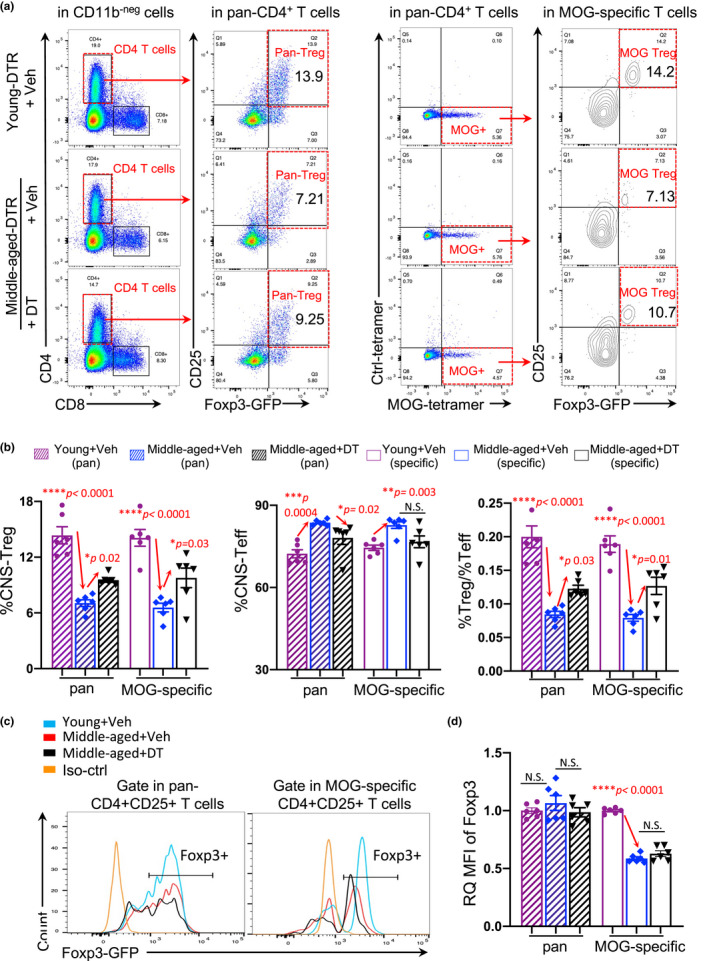
Transient depletion of the accumulated pTreg cells improved Treg CNS trafficking in middle‐aged *FoxP3* ^DTR^ EAE mice. (a) Flow cytometry gating strategies of pan‐Treg and MOG‐specific Treg cells in the CNS of young and middle‐aged EAE mice with DT or vehicle treatment. (b) Summarized results of the percentages of pan‐ (striped bars) and MOG‐specific (opened bars) CNS‐Treg (left panel) and CNS‐Teff (middle panel) cells, and ratios of Treg/Teff cells (right panel) in the CNS of young (cherry) and middle‐aged EAE mice treated with vehicle (blue) and DT (black), respectively. (c) Flow cytometry gating strategies show representative FoxP3^+^ gates from CD4^+^CD25^+^ gates of the CNS of the three groups of EAE mice. (d) Summarized results of the RQ‐MFI of FoxP3 expression in pan‐ (striped bars) and MOG‐specific (open bars) CD4^+^CD25^+^ population in the CNS of young (cherry) and middle‐aged EAE mice treated with vehicle (blue) or DT (black). All data are expressed as mean ± SEM and are analyzed by the one‐way ANOVA followed by the Dunnett's multiple post hoc test, and a statistically significant difference was considered to be *p* < 0.05, “N.S.” stands for “not significant”

One day after the last treatment with P300i in aged mice (Figure 5a, the 2nd test red arrow), we found that the expression of IFN‐γ was increased in the CP‐adherent hematopoietic CD45^+^ cells (Figure 6a black line in the pink box, and right table). We also determined the impact on CNS‐infiltration of Treg cells after transient inhibition of pan‐pTreg cells, showing that both the percentages of CNS pan‐Treg cells (Figure 6b left panel and c black striped bar in left panel) and CNS MOG‐sp. Treg cells (Figure 6b right panel and c black open bar in left panel) were increased in the inflamed CNS of aged mice. The lack of change in FoxP3 expression in CNS‐infiltrated Treg cells (Figure 6d,e) between mice with and without transient inhibition may indicate that the drug P300i does not affect CNS‐infiltrated Treg cell function. In addition, increased CNS‐Treg cells could either suppress CNS‐Teff cells (Figure 6c middle panel) or increase the Treg‐to‐Teff ratio in the inflamed CNS (Figure 6c right panel). This ratio was observed to be imbalanced during late‐onset EAE disease (Figure 2e right panel). Likewise, this partially restored Treg‐to‐Teff balance in the aged EAE CNS was also observed in the middle‐aged DTR EAE mice with the transient pTreg depletion (Figure 7). In addition, Th1 (IFN‐γ^+^) and Th17 (IL‐17A^+^) cytokine production was inhibited in pan‐ and MOG‐sp. CD4^+^ T cells (Figure S8a,b) along with the reduced proinflammatory cytokine production by CD11b^+^ myeloid immune cells (Figure S8c,d) in the CNS of DT‐treated middle‐aged DTR mice, which is associated with alleviated proinflammatory conditions in the aged EAE CNS.

These results suggest that the potential underlying mechanism for mitigation of late‐onset EAE severity via the transient inhibition or depletion of the accumulated pan‐pTreg cells is to increase INF‐γ produced by the CD45^+^ leukocytes residing at the CP and facilitate the trafficking of immune cells into the aged, inflamed CNS (Raposo et al., 2014), including not only pan‐ and MOG‐sp. Treg cells, but also likely other anti‐inflammatory monocytes, such as monocyte‐derived anti‐inflammatory cells, to reduce the CNS inflammation. This is very similar to the mechanism shown with this method to mitigate AD pathology (Baruch et al., 2015).

## 3 DISCUSSION

Typically, human MS disease develops in young (20–40 years old) women and presents with at least four clinical subtypes. Two of them, progressive‐relapsing MS (PRMS) and RRMS, have relatively mild symptoms, and are often seen in young patients (80%–90%). However, the other two, PPMS and SPMS, have severe symptoms and are often seen in aged (>65 years old) patients (~29% for PPMS and ~26% for SPMS), and young patients only account for 10% of PPMS and rarely exhibit SPMS (Sanai et al., 2016). Late‐onset MS in the elderly has been reported (Bermel et al., 2010; Polliack et al., 2001; Sanai et al., 2016), and the mean age of the MS population is rising (Sanai et al., 2016). It was reported that 14% of total MS patients were 65 years and older in 2010 (Awad & Stuve, 2010). The severe symptoms in the aged patients are more or less attributed to Treg cell function, since Treg cells are regarded as autoimmune protective cells (Buckner, 2010; Furtado et al., 2001; Lowther & Hafler, 2012), which combat IFN‐γ‐producing and IL‐17‐producing CD4^+^ pathogenic cells and pathogenic CD8^+^ T cells involved in the MS lesion.

Ample evidence shows that Treg cells play an ameliorative role in MS/EAE disease onset and severity (Buc, 2013; Furtado et al., 2001; Kleinewietfeld & Hafler, 2014). Transferring Treg cells into MOG‐induced EAE mice attenuated disease (Kohm et al., 2002) and EAE severity correlated inversely with the frequency of MOG‐sp. Treg cells (Reddy et al., 2004). However, the accumulation of pTreg cells (Raynor et al., 2012) with enhanced suppressive function (Garg et al., 2014) in aged individuals is accompanied by severe MS/EAE symptoms in aged humans (Sanai et al., 2016) and mice (Figure 1). This is not consistent with the notion that Treg cells play a role in suppressing uncontrolled immune reactions. This inconsistency encouraged us to investigate the underlying mechanism.

CD4^+^FoxP3^+^ Treg cells primarily act to suppress Teff cell‐mediated aberrant antigen‐specific and nonspecific immune responses. Accumulation of pTreg cells in the elderly is disadvantageous for fighting infection, cancer, and neurodegenerative diseases. The notion is that excess Treg cells may even be harmful. In protection and recovery from CNS inflammatory disorders, such as AD, one of the age‐related neuroinflammatory diseases, excessive pTreg cells were shown to play a detrimental role (Baruch et al., 2015). This is probably due to Treg cell distribution either inside the CNS or in the periphery (Coder et al., 2016), in addition to the existence of distinct Treg subsets (Liu et al., 2014; Saresella et al., 2010). Herein, using an aged EAE mouse model that resembles aged human MS disease, we found that aged EAE mice had a different distribution of pan‐ and MOG‐sp. Treg cells compared with young counterparts. Specifically, aged mice exhibited a high proportion of pan‐pTreg and a low proportion of MOG‐sp. pTreg cells in their periphery but low proportions of both pan‐ and MOG‐sp. CNS‐Treg cells in the inflamed CNS (Figure 2). Accumulation of pTreg cells outside the CNS and residing at the CNS‐periphery boundaries, the BBB and CP, could impede immune cells, including Treg cells, trafficking into the inflamed CNS for recovery (Baruch et al., 2015; Raposo et al., 2014).

Treg cells are unstable and plastic, and this is especially observable during inflammatory autoimmune stimulation (Bailey‐Bucktrout et al., 2013; Dominguez‐Villar & Hafler, 2018). The unstable characteristics are exhibited by loss of FoxP3 expression in a proportion of mature Treg cells (Zhou et al., 2009), and the plastic characteristics are exhibited by the production of proinflammatory cytokines IFN‐γ or IL‐17, along with FoxP3 expression. These Treg cells acquire an effector‐like phenotype, and they can induce, rather than suppress, autoimmunity. These cells are commonly seen in autoimmune‐prone NOD mice and diabetic patients (McClymont et al., 2011; Tan et al., 2016), and in MS patients (mouse EAE settingKitz et al., 2016; Korn et al., 2007). We found that in the aged, inflamed CNS‐Treg cells exhibited higher plasticity than in young counterparts, observed as the co‐expression of INF‐γ and/or IL‐17 with FoxP3 (Figure 3c), which potentially results in reduced suppressive function and increased pathology (Dominguez‐Villar & Hafler, 2018; Kitz & Dominguez‐Villar, 2017). Further, we observed differences in CNS‐Teff clonal expansion, which shed light on the reduced suppressive function of CNS‐infiltrated Treg cells in aged mice (Figure 4a, top pies). While the clonal expansion of CNS‐infiltrated Treg cells was almost the same between young and aged EAE mice, the clonal expansion of CNS‐Teff cells was significantly increased in the aged EAE mice. In addition, the lower Treg clonal expansion reflects their biological feature. Our data also showed that expanded CNS‐infiltrated Treg clones (clone size ≥2) are only accounting for 3%–4% of total CNS‐infiltrated Treg clones in either young or aged mice (Figure 4a, bottom pie charts). The higher clonal expansion of CNS‐Teff cells in aged, inflamed CNS could explain the lower suppressive function of the CNS‐Treg cells, since this reflects a disruption of the normal Treg/Teff balance. In addition to self‐antigen‐driven inflammatory autoimmune stimulation (Bailey‐Bucktrout et al., 2013; Dominguez‐Villar & Hafler, 2018), the age‐related, chronic, systemic inflammation (inflammaging) may play a synergistic role to enhance CNS‐Treg plasticity in aged EAE mice compared with young counterparts. However, this synergistic role needs further investigation. One technical limitation is that we were not able to identify the clonal expansion of MOG‐sp. T cells among the whole T cell population using sc‐RNA‐Seq, since a unique sequence‐based MHC‐II MOG‐Dextramer reagent is under development.

It is a difficult and complex task to mitigate the accumulation of pTreg cells in the elderly as a therapeutic strategy for the neuroinflammatory disease. Transient inhibition of FoxP3 expression in the accumulated peripheral CD4^+^FoxP3^+^ Treg cells in aged individuals is one option, which was used in AD and displayed improvement of amyloid‐beta plaque clearance, amelioration of neuroinflammation, and recovery of cognitive decline demonstrated in an AD mouse model (Baruch et al., 2015). We transiently inhibited or depleted accumulated pTreg cells in aged mice, which significantly ameliorated EAE disease and corrected Treg distribution (Figures 5, 6, 7). The mechanism is likely the accumulated CP‐resident Treg cells suppressing INF‐γ producing cells, such as Th1 CD4^+^ T cells outside the CNS, which directly block the gateway (Baruch et al., 2015) for homeostatic leukocyte trafficking into the CNS (Deczkowska et al., 2016; Kunis et al., 2013). IFN‐γ is required for activation of the brain's CP for CNS immune surveillance and repair (Kunis et al., 2013; Raposo et al., 2014). For example, immunization with a myelin‐derived antigen was reported to activate the CP via inducing the CP to express IFN‐γ and attract Th1 cells, thereby enhancing recruitment of immunoregulatory cells to the CNS to achieve attenuation of neuroinflammatory progression in a mouse model (Kunis et al., 2015).

Although CD4^+^ T cells, both CD4^+^ Teff and CD4^+^FoxP3^+^ Treg cells, are the traditional primary actors in MS/EAE pathogenesis and immunoregulation, emerging evidence shows that CD8^+^ T cells also contribute to immunopathology and immunoregulation and can either exacerbate or mitigate brain inflammation during CNS autoimmunity (Mars et al., 2011; Mockus et al., 2021). Regarding MS/EAE immunopathogenesis, myelin‐specific CD8^+^ T cells exacerbate brain, though not the spinal cord, inflammation via a Fas ligand‐dependent mechanism to promote lesion formation in the brain (Wagner et al., 2020). In addition, IL‐17A secreting CD8^+^ T cells, termed Tc17 cells, in the CNS support Th17 cell‐mediated autoimmune encephalomyelitis (Huber et al., 2013; Saxena et al., 2012). In aged neurogenic niches that comprise neural stem cells, CD8^+^ T cells are increased and inhibit the proliferation of neural stem cells (Dulken et al., 2019). This is a disadvantage for the recovery of demyelinating lesions in aged MS/EAE disease. Our results show that Tc17^+^ cells were increased in the aged EAE CNS (Figure 3d), which was consistent with severe symptoms and pathology in aged EAE mice. However, regarding MS/EAE immunoregulation, although the concept of CD8^+^Treg cells is not unanimously accepted, the function of CD8^+^Treg cells in MS/EAE has received attention (Baughman et al., 2011; Niederlova et al., 2021; Sinha et al., 2014). The main focus of studies of CD8^+^Treg cells in MS/EAE is young patients or young animals, and there are many unanswered questions regarding how these cells contribute to CNS autoimmune inflammation in the elderly. Therefore, the function of these cells in the elderly needs to be investigated in future.

In summary, the results herein provide insights into how accumulated aged polyclonal CD4^+^FoxP3^+^ pTreg cells in an inflammatory condition do not ameliorate but are detrimental to CNS repair processes in neuronal inflammation of aged MS demonstrated in the animal model EAE.

## 4 METHODS

### 4.1 Mice and animal care

C57BL/6 wild‐type (WT) mice were used. Aged (18–20 months old) mice were ordered from the National Institute on Aging, aged rodent colonies. Control young WT mice were 2–3 months old. FoxP3‐DTR/GFP mice (young 2 months old, middle‐aged 12–15 months old) were obtained from the Jackson Laboratory (Stock No: 016958). All mice were maintained under specific pathogen‐free conditions in the animal facilities at the University of North Texas Health Science Center. All animal experiments were performed in compliance with protocols approved by the Institutional Animal Care and Use Committee of the University of North Texas Health Science Center (IACUC‐2018‐0014 and 2021‐0020), in accordance with guidelines of the National Institutes of Health.

### 4.2 Late‐onset EAE mouse model and disease score determination

EAE was induced as shown in Figure 1a. Briefly, MOG_35‐55_ peptide (WatsonBio Sciences) was emulsified in Complete Freund′s Adjuvant (Sigma‐Aldrich F5881) and given as one‐time subcutaneous (s.c.) injection into the upper and lower backs of mice (80 µg MOG_35‐55_ peptide/10 g body weight). Pertussis toxin (PT; List Biologicals, Cat#179A) was intraperitoneally (i.p.) injected on days 0 and 1 (100 ng PT/10 g body weight). Mice were monitored daily for EAE symptoms and body weight changes. EAE scores were assigned in Table S1.

### 4.3 Transient inhibition or partial depletion of pan‐pTreg cells in aged mice

WT young and aged mice, as shown in Figure 5a, 12 days after immunization (DPI), received P300i (C646; Tocris Bioscience, Cat# 4200) i.p. injection at 8.9 mg/kg body weight per day for 5 consecutive days (5x) in one of the aged mouse groups similar to a published protocol (Baruch et al., 2015). Vehicle‐injected young and aged mice were controls. The dynamic changes of Treg cell frequencies and Foxp3 expression were monitored at three time‐points as denoted by red arrows in Figure 5a. To *FoxP3* ^DTR^ mice, 12‐DPI in young and middle‐aged DTR mice, diphtheria toxin (DT; Sigma‐Aldrich, Cat#D0564) was i.p. injected at 50 μg/kg body weight to one of the aged DTR mouse groups. Vehicle injections were given to controls.

### 4.4 Cell isolation from the CNS and the brain CP

For mononuclear cell isolation from the brains and the spinal cords, euthanized mice were transcardially perfused with 20ml of PBS. CNS tissues were minced and digested with 2mg/ml collagenase‐D (Roche, Cat# 11088858001) and 28 U/ml DNase I (Invitrogen, Cat# 18068015) in RPMI‐1640 at 37°C for 45 min, followed by percoll (Sigma‐Aldrich, Cat# P1644) gradient centrifugation per our previous method (Wang et al., 2018). For cell isolation from the brain CP, CP tissues (5 mice per group) were collected from the lateral, third, and fourth ventricles of the brain then digested at 37°C for 40 min in 2 mg/ml collagenase‐D/PBS with pipetting.

### 4.5 Tetramer‐based flow cytometric assay of pan‐ and MOG‐specific Treg and Teff cells, cytokine productions by CNS immune cells, and IFN‐γ producing cells at the CP

Single‐cell suspensions from the LN, spleen, and the CNS of mice were stained with extracellular fluorochrome‐conjugated CD antibodies (Biolegend), along with APC‐conjugated MOG_38‐49_ I‐A^b^ tetramer (NIH tetramer core, peptide sequence: GWYRSPFSRVVH) and Brilliant Violet 421‐conjugated human CLIP_87‐101_ I‐A^b^ tetramer as a control (peptide sequence: PVSKMRMATPLLMQA). Then, cells were fixed and permeabilized using the kit (eBioscience, Cat# 00‐5523‐00) for intracellular staining of PE‐conjugated anti‐FoxP3 (eBioscience Cat# 12‐5773‐82) per the instruction. For intracellular staining of CNS isolated immune cells, cells were stimulated with PMA (5 ng/ml), ionomycin (500 ng/ml), and protein transport inhibitor (0.7 µl/ml, BD Biosciences. Cat# 51‐2092KZ) for 5hrs, followed by extracellular marker and intracellular cytokine antibody staining. Data were analyzed using FlowJo™ v10 software. Mean fluorescence intensity (MFI) was defined as the “medians” of fluorescence intensities of the conjugated fluorochromes of the antibodies.

Cells isolated from the CP were incubated with PMA, ionomycin, and protein transport inhibitor as above for 5 h, followed by extracellular staining with PerCP/Cy5.5 conjugated anti‐CD45 (Biolegend, Cat# 103132) and intracellular staining with APC‐conjugated anti‐IFN‐γ (Biolegend, Cat# 505810).

### 4.6 ScRNA‐seq of CNS T cells for analysis of transcriptome profile and TCR copy number‐based T cell clonal expansion

Mononuclear cells were isolated from the CNS of three young and three aged EAE mice. Cells were stained with anti‐CD3‐APC (Biolegend, Cat#100236) and anti‐CD11b‐Brilliant Violet 711 (Biolegend, Cat#101242), then sorted on Sony SH800 Cell Sorter to collect CD3^+^CD11b^−neg^ T cells, followed by gene expression (GEX) and TCR V(D)J library preparation and sequencing with the 10x Genomics Chromium Single Cell 5ʹ GEM, Library & Gel Bead Kit v2 (10x Genomics, Cat#1000287). The cDNA was amplified using the same kit. Products were purified using Ampure XP beads, and quality was controlled using Agilent Tapestation and Qubit 4 fluorometer. TCR target enrichment was done by Chromium Single Cell Mouse TCR Amplification kit (10x Genomics, Cat #1000254). TCR V(D)J, and GEX libraries were constructed by the Library Construction Kit (10x Genomics, Cat #1000190) with Dual Index Kit TT Set A. Sequencing was on an Illumina NovaSeq 6000 according to 10x Genomics sequencing protocol recommendations.

Fastq files (Cell Ranger, version 6.0.2, 10xGenomics provided mm10 reference genome), Cloupe files, and Vloupe files were generated for the downstream analysis. The t‐distributed stochastic neighbor embedding (t‐SNE) plots of T cells were visualized by 10x Genomics Loupe Brower 5.0, and T cells were classified into three groups, including CD8 T cells, CD4 Teff cells, and CD4 Treg cells. Normalized feature expressions of *Ifng* and *Il17a* were used to determine the gene expression of CNS‐infiltrated CD4^+^ Treg, CD4^+^ Teff, and CD8^+^ T cell populations at the single‐cell level. The R packages Seurat and clusterProfiler were used for gene expression matrix generation and GO enrichment analysis. 10x Genomics Loupe VDJ Brower 4.0 was used to output the clonotypes of CD4^+^ Teff and Treg cells by aggregating the Cloupe file and Vloupe file of each sample. Each clone was determined by the CDR3 regions of paired TCRα and TCRβ chains. Based on TCR sequence similarity, clones with clonal size greater than 2 were defined as expanded clones, among which the most frequent 10 clones in each sample were defined as the top 10 expanded clones. All unique clones were defined as unexpanded clones.

### 4.7 Luxol Fast Blue staining for demyelination assay of the spinal cords

Paraffin sections of the spinal cord (Figure 5d) were stained with 0.1% Luxol Fast Blue (LFB) solution (Sigma‐Aldrich, Cat#S3382) per a previous publication (Yoo et al., 2019), with a modification by adding eosin counterstaining (details in Supplemental Method‐B).

### 4.8 Statistics

Statistical tests used to analyze each set of experiments are indicated in each figure legend, including the unpaired two‐tailed Student's *t*‐test for two groups and one‐way ANOVA for multiple groups. Data from EAE scoring were analyzed by the Mann–Whitney *U* test to compare between two groups, and the Kruskal–Wallis test was used to compare multiple groups followed by the Dunnett's multiple comparisons post hoc test for pairwise comparisons of groups. Body weight changes over time between two groups were analyzed with two‐way repeated‐measures ANOVA with Geisser‐Greenhouse correction. Results were considered statistically significant at values of *p < 0.05; **p < 0.01; ***p < 0.001; ****p < 0.0001.

## AUTHOR CONTRIBUTIONS

W.W. performed most of the experiments and hands‐on animal work, and wrote the manuscript; R.T. assisted with cell sorting experiments and edited the manuscript; J.O. performed some experiments; D.‐M.S. conceived and designed the project, did some hands‐on animal work, analyzed the data, and wrote the manuscript.

## CONFLICT OF INTEREST

The authors declare no competing interests.

## Supporting information

Fig S1‐S8‐Table S1

Supporting Source Data File

## Data Availability

All sc‐RNA‐seq raw data are available on the Gene Expression Omnibus (GEO) (GSE182747) [https://www.ncbi.nlm.nih.gov/geo/query/acc.cgi?acc=GSE182747]. All individual numerical values, detailed statistic information, and other regarding source data in each figure are included in Supporting Source Data File.xlsx.
