# Supplementary material for: Accumulation of pTreg cells is detrimental in late‐onset (aged) mouse model of multiple sclerosis
Source: Aging Cell. 2022 May 26;21(6):e13630. doi: 10.1111/acel.13630 (PMC9197401; doi:10.1111/acel.13630)
Supplement: Supplementary file 1 — Fig S1‐S8‐Table S1 [file ACEL-21-e13630-s001.pdf]

A.

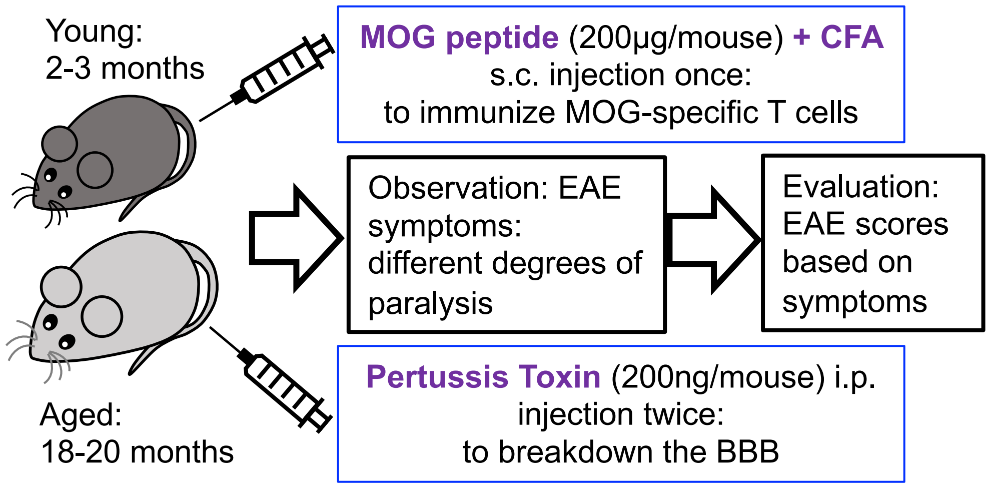

B.

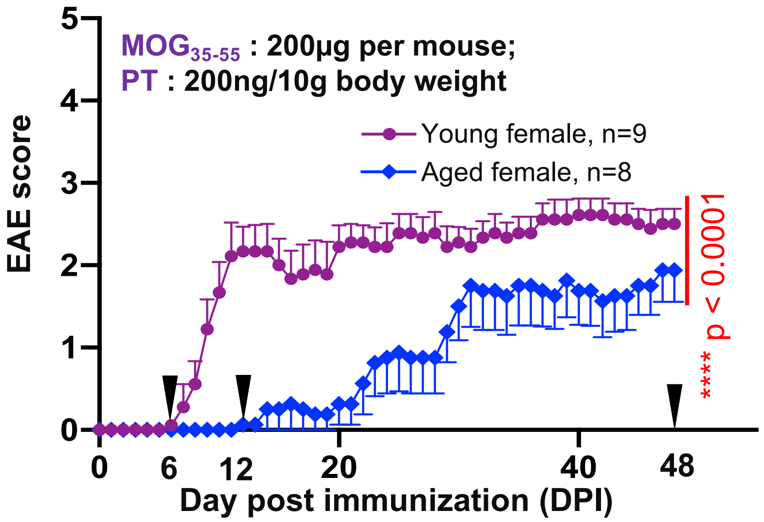

Supplemental Figure S1. Establishment of late-onset (aged) mouse EAE model for aged human MS using a standard immunization protocol. (A) Workflow for EAE induction in young and aged C57BL/6 female mice using a standard immunization protocol, followed with daily EAE score evaluation. **(B)** Characteristics of EAE pathological scores in young (cherry-color line) versus aged (blue line) female EAE mice. The results indicate that with per mouse-based immunization dosage, most aged mice showed a delayed onset (12 days post immunization, DPI) compared to the young (6-DPI). 8 out of 13 (~2/3) of the aged mice showed no disease or very mild symptoms, and never reached a debility score >3.0 by 48-DPI (blue line). This observation is contradictory to the increased symptom severity and progression in aged MS patients.

C57BL/6 mouse age-body weight information (from the Jackson laboratory):
https://www.jax.org/jax-mice-and-services/strain-data-sheet-pages/body-weight-chart-000664#
https://www.jax.org/jax-mice-and-services/strain-data-sheet-pages/body-weight-chart-aged-b6

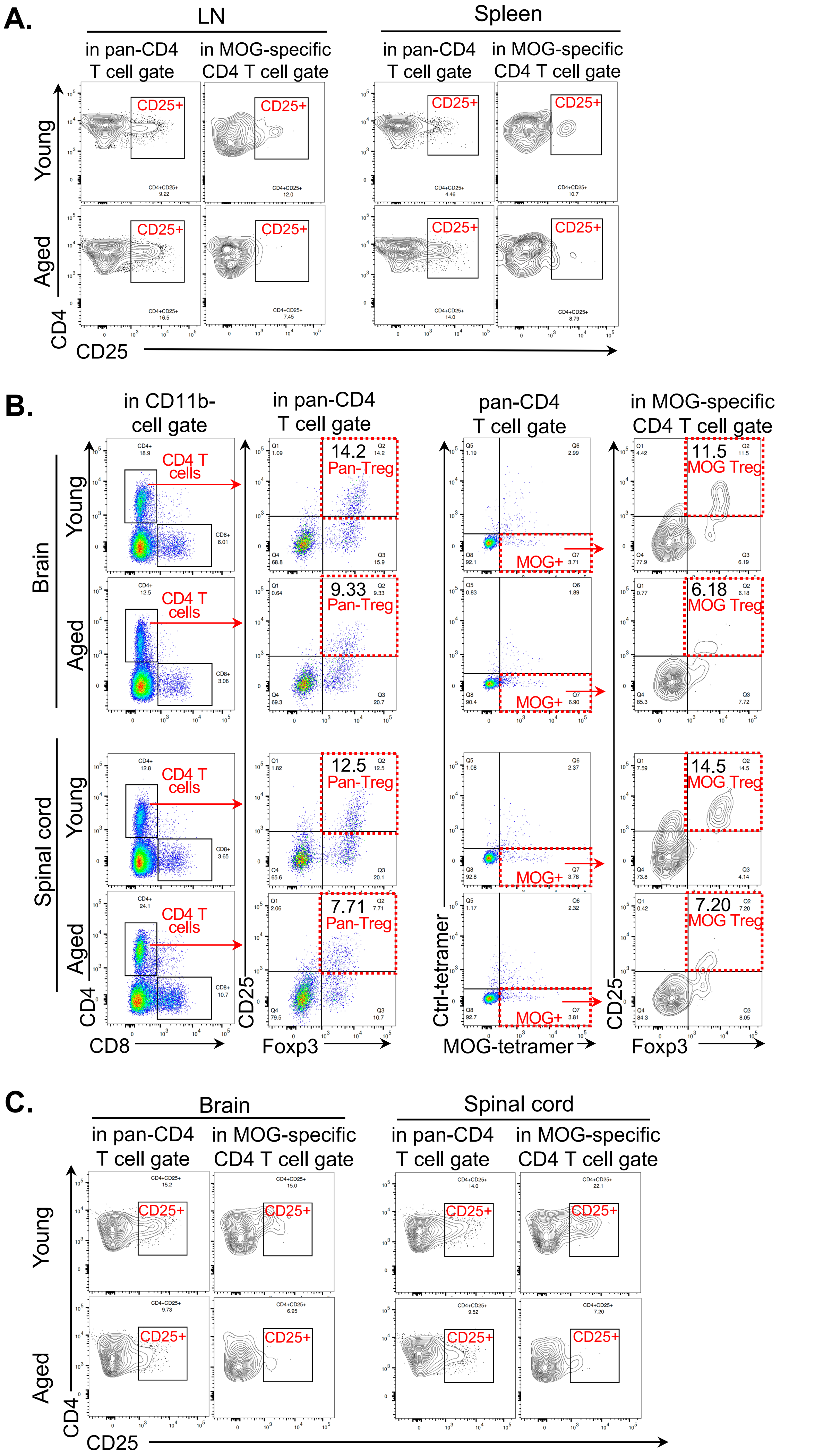

Supplemental Figure S2. Gating strategies for CD4⁺CD25⁺ population for FoxP3 MFI analysis and for CNS pan- and MOG-sp. Treg cells analysis in young and aged EAE mice. (A) Representative gating strategies of CD25⁺ populations in CD4⁺ pan-T cells and CD4⁺ MOG-sp. T cells for FoxP3 MFI analysis of the spleen and LN (Detailed elaboration of Fig. 2C). **(B)** Representative gating strategies of pan-CNS-Treg cells and MOG-sp. CNS-Treg cells in young and aged EAE mice (Detailed elaboration of Fig. 2E). **(C)** Representative gating strategies of CD25⁺ populations in CD4⁺ pan-T cells and CD4⁺ MOG-sp. T cells for CNS-Treg FoxP3 MFI analysis of the brain and spinal cord (Detailed elaboration of Fig. 2F).

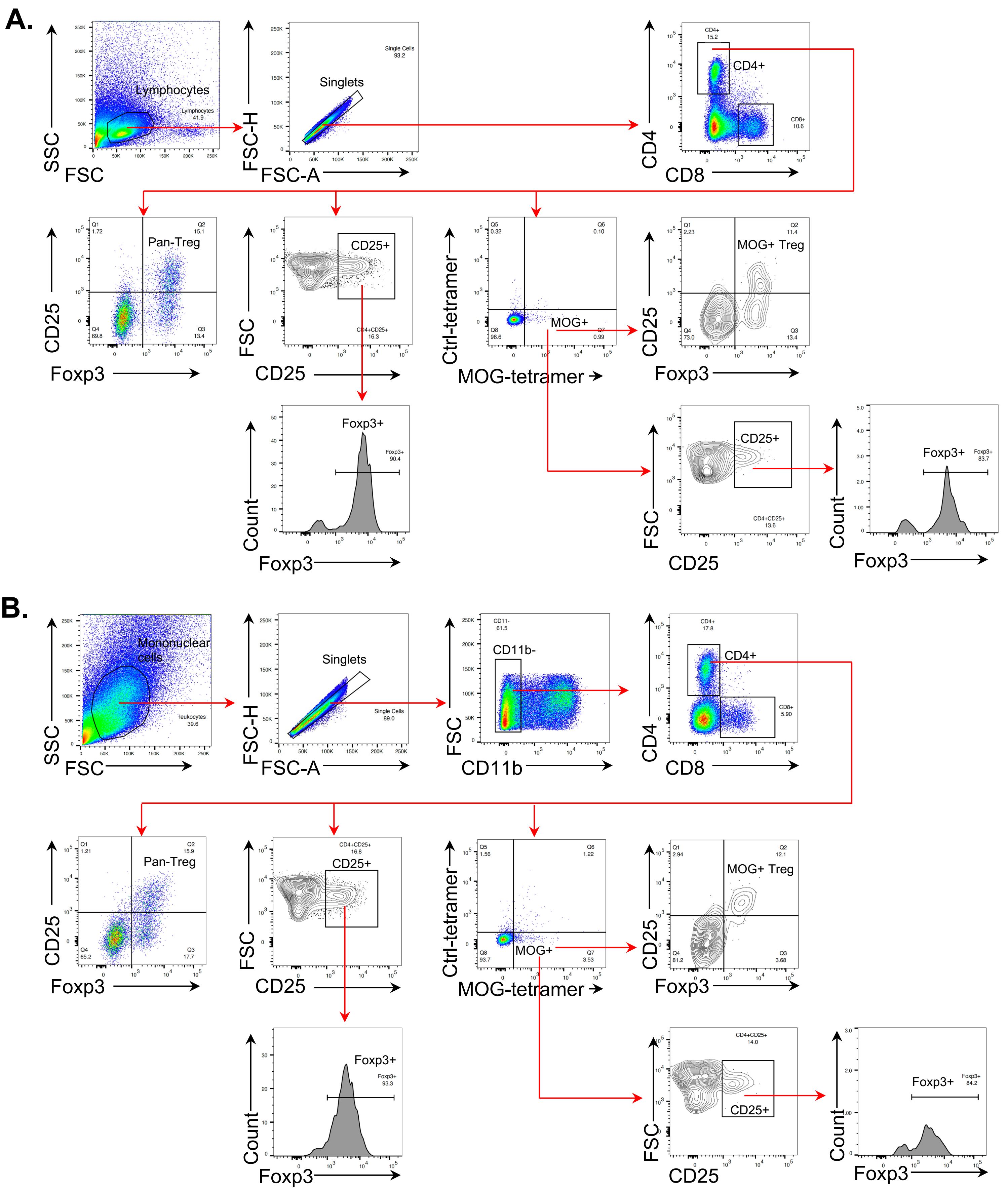

Supplemental Figure S3. Representative Flow cytometric gating strategies of T cell populations in the periphery (A) and CNS (B) for Figure 2.

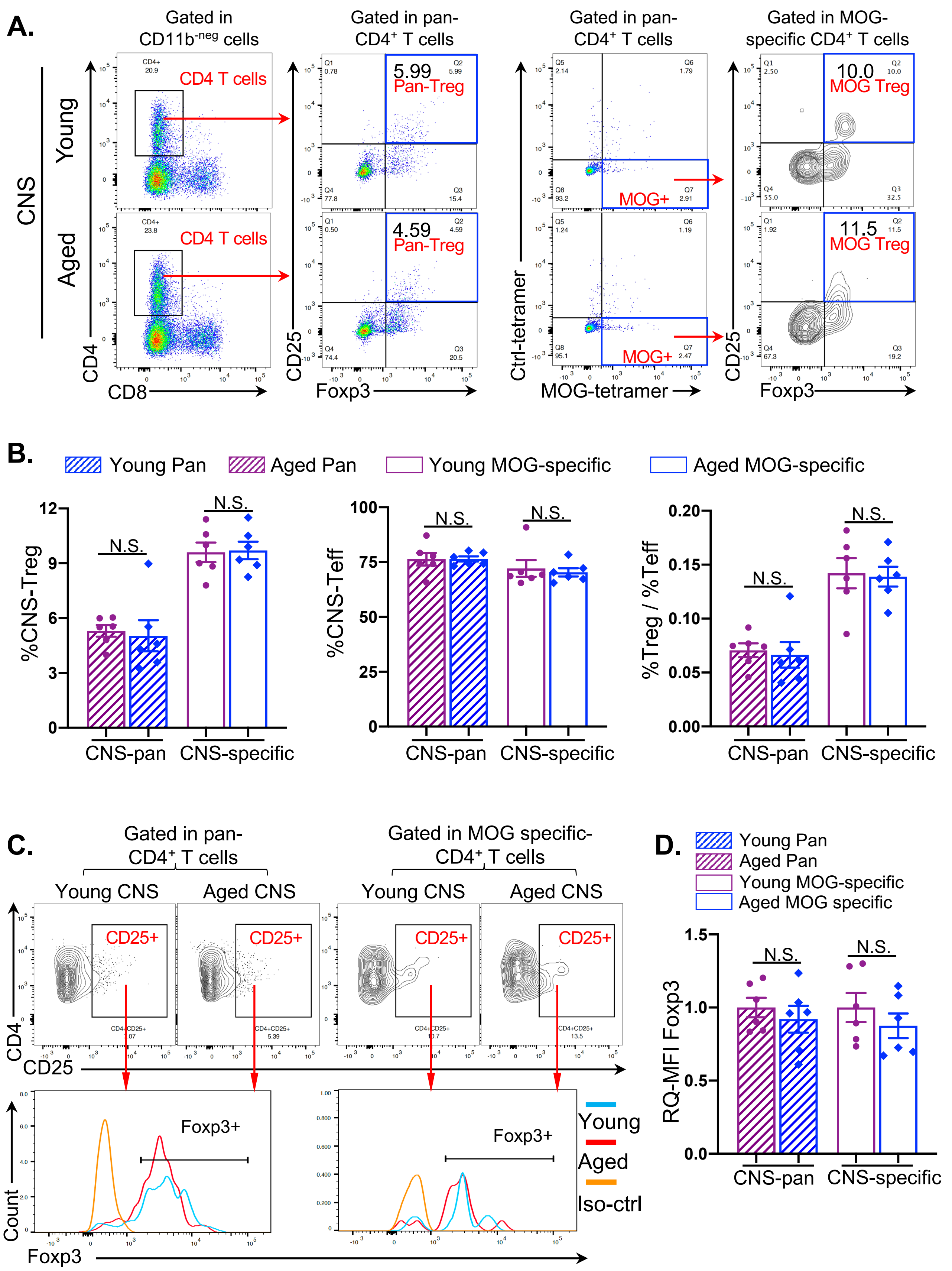

Supplemental Figure S4. Distribution of pan- and MOG-specific CNS-Treg in the CNS of young and aged mice at early disease stage (on 8-DPI). (A) Representative flow cytometry gating strategies of pan- and MOG-specific CNS-Treg cells of young and aged mice. **(B)** Summarized results of the percentages of pan- and MOG- specific CNS-Treg cells and CNS-Teff cells, and Treg/Teff ratio of young and aged mice. **(C)** Representative flow cytometry gating strategies show $FoxP3^+$ gates from $CD4^+CD25^+$ gates of the CNS of young and aged mice. **(D)** Summarized results of the RQ-MFI of FoxP3 expression in pan-and MOG-specific CNS $CD4^+CD25^+$ population in young and aged mice.

The results indicate that a discrepancy of CNS-Treg cells between the young and aged mice was not observed at early stage of EAE onset before noticeable symptoms. This may suggest that the imbalanced CNS-Treg distribution in the aged EAE mice is associated with severity of EAE disease.

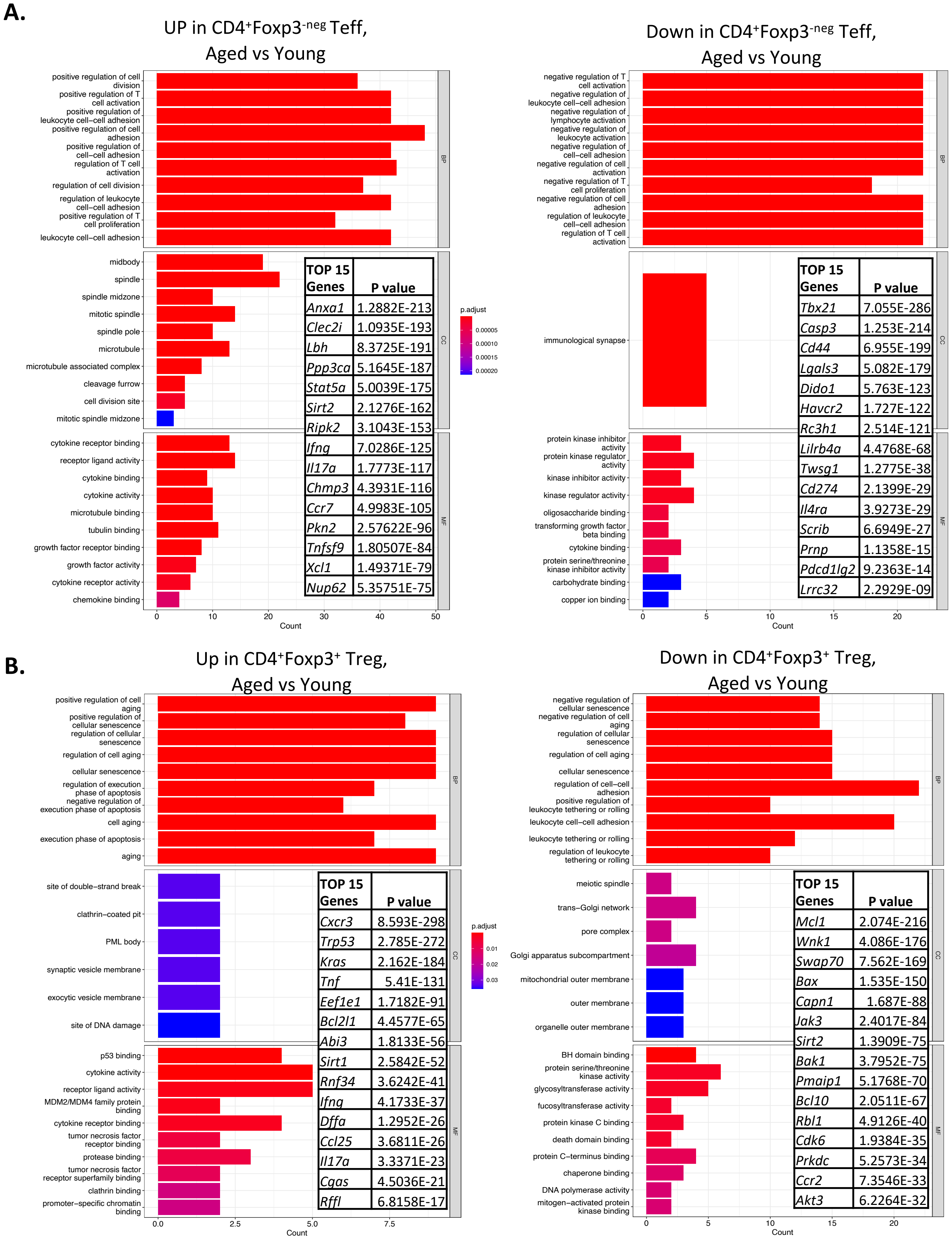

A. UP in CD4+Foxp3-neg Teff, Aged vs Young

TOP 15 Genes	P value
Anxa1	1.2882E-213
Clec2i	1.0935E-193
Lbh	8.3725E-191
Ppp3ca	5.1645E-187
Stat5a	5.0039E-175
Sirt2	2.1276E-162
Ripk2	3.1043E-153
Ifng	7.0286E-125
Il17a	1.7773E-117
Chmp3	4.3931E-116
Ccr7	4.9983E-105
Pkn2	2.57622E-96
Tnfsf9	1.80507E-84
Xcl1	1.49371E-79
Nup62	5.35751E-75

A. Down in CD4+Foxp3-neg Teff, Aged vs Young

TOP 15 Genes	P value
Tbx21	7.055E-286
Casp3	1.253E-214
Cd44	6.955E-199
Lgals3	5.082E-179
Dido1	5.763E-123
Havcr2	1.727E-122
Rc3h1	2.514E-121
Lilrb4a	4.4768E-68
Twsg1	1.2775E-38
Cd274	2.1399E-29
Il4ra	3.9273E-29
Scrib	6.6949E-27
Prnp	1.1358E-15
Pdcd1lg2	9.2363E-14
Lrrc32	2.2929E-09

B. Up in CD4+Foxp3+ Treg, Aged vs Young

TOP 15 Genes	P value
Cxcr3	8.593E-298
Trp53	2.785E-272
Kras	2.162E-184
Tnf	5.41E-131
Eef1e1	1.7182E-91
Bcl2l1	4.4577E-65
Abi3	1.8133E-56
Sirt1	2.5842E-52
Rnf34	3.6242E-41
Ifng	4.1733E-37
Dffa	1.2952E-26
Ccl25	3.6811E-26
Il17a	3.3371E-23
Cgas	4.5036E-21
Rffl	6.8158E-17

B. Down in CD4+Foxp3+ Treg, Aged vs Young

TOP 15 Genes	P value
Mcl1	2.074E-216
Wnk1	4.086E-176
Swap70	7.562E-169
Bax	1.535E-150
Capn1	1.687E-88
Jak3	2.4017E-84
Sirt2	1.3909E-75
Bak1	3.7952E-75
Pmaip1	5.1768E-70
Bcl10	2.0511E-67
Rbl1	4.9126E-40
Cdk6	1.9384E-35
Prkdc	5.2573E-34
Ccr2	7.3546E-33
Akt3	6.2264E-32

Supplemental Figure S5. Gene ontology (GO) enrichment analysis of up-regulated (left) and down-regulated (right) gene sets in aged CD4^{+} CNS-Teff cells and CD4^{+} CNS-Treg cells. (A) GO enrichment analysis of up-regulated (left panel) and down-regulated (right panel) gene enriched pathways in aged CD4^{+}Foxp3^{-neg} CNS-Teff cells with the top 15 significantly upregulated and downregulated genes (inserted boxes in the left and right panels) of aged CD4^{+}Foxp3^{-neg} CNS-Teff cells. **(B)** Same analysis as in (A) of aged CD4^{+}Foxp3^{+} CNS-Treg cells.
The results imply that aged Treg cells, which have infiltrated into the CNS after EAE onset, could have reduced capacity to suppress CNS-Teff cell-induced neuronal inflammation.

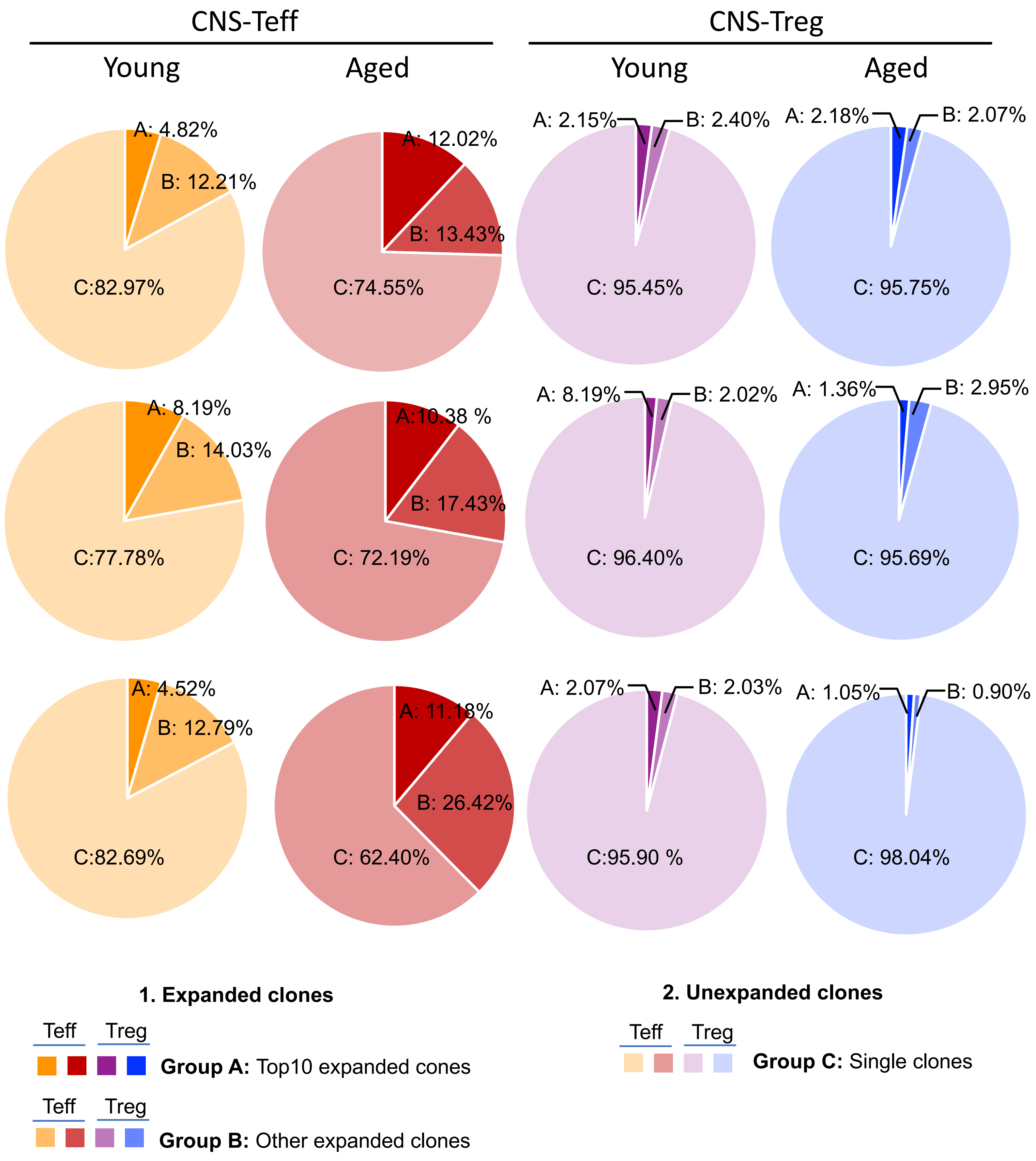

Supplemental Figure S6. Clonal expansion in CNS-infiltrated CD4$^+$ T cells of three young and three aged EAE mice. Leftmost column: three individual pie charts of young CNS-Teff cell clonal expansion; second left column: three individual pie charts of aged Teff cell clonal expansion; second right column: three individual pie charts of young Treg cell clonal expansion; rightmost column: three individual pie charts of aged Treg cell clonal expansion. The results are for detailed elaboration of Fig. 4.

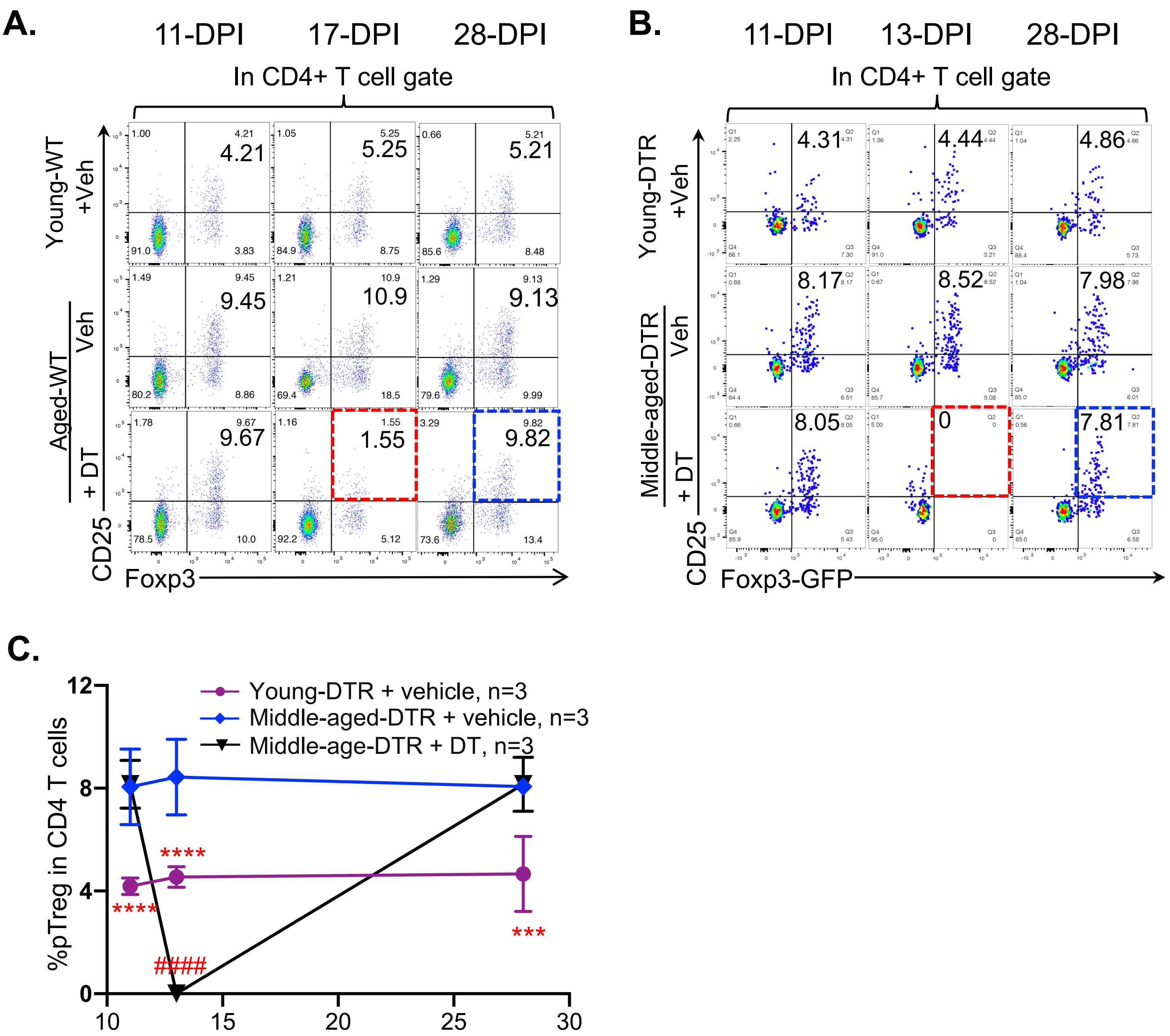

Supplemental Figure S7. Gating strategies of time-course changes in peripheral blood pTreg cells after the transient inhibition or depletion. (A) WT EAE mice; **(B)** and **(C)** *FoxP3*^{DTR} EAE mice. **(A)** and **(B)** Flow cytometry gating strategies show the frequencies of peripheral blood Treg cells in the three groups of mice 11-DPI (one day before p300i treatment in A or DT-treatment in B), 17-DPI in A or 13-DPI in B (one day after the last p300i or one day after DT injection), and 28-DPI (12 days after the last p300i or 16 days after DT treatment). Red box shows reduction and blue box shows recovery of pTreg cells. **(C)** Summarized results of time-course changes of pTreg frequencies in the peripheral blood of three groups of *FoxP3*^{DTR} EAE mice. The results are for detailed elaboration of Fig. 5.

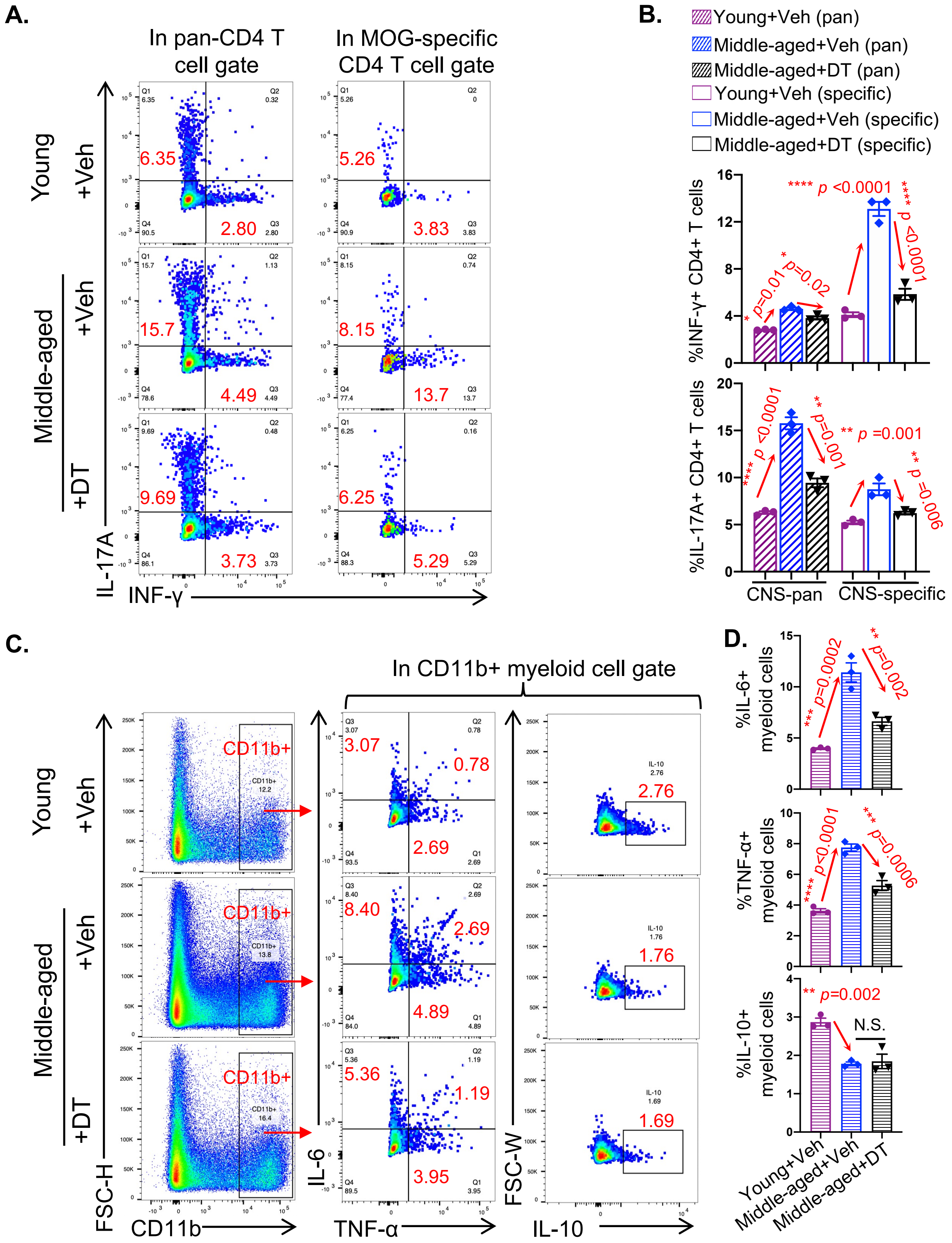

Supplemental Figure S8. Inflammatory cytokine productions by CNS T cells and myeloid cells after transient depletion of pTreg cells in middle-aged *FoxP3*^{DTR} EAE mice. (A) Flow cytometric gating strategies of INF-γ^{+} and IL-17A^{+} pan-CD4 T cells and MOG-specific CD4 T cells in EAE CNS of young and middle-aged *FoxP3*^{DTR} mice with or without DT treatment. **(B)** Summarized results of the percentages of pan- and MOG-specific INF-γ^{+} and IL-17A^{+} CNS-CD4 T cells in the EAE CNS of young and middle-aged mice, treated with vehicle or with DT. **(C)** flow cytometric gating strategies of IL-6^{+}, TNF-α^{+} and IL-10^{+} myeloid immune cells in the EAE CNS of young and middle-aged *FoxP3*^{DTR} mice with or without DT treatment. **(D)** Summarized results of the percentages of IL-6^{+} , TNF-α^{+} and IL-10^{+} myeloid immune cells in the EAE CNS of young and middle-aged mice treated with vehicle or with DT. The results display that transient depletion of accumulated pTreg cells in middle-aged mice results in reduction of proinflammatory cytokines in the inflamed CNS.

Supplemental Methods:

A. Table S1: Mouse EAE scoring

Score	Clinic observations
0	No obvious changes in motor function compared to unimmunized mice
0.5	tail tip limpness
1.0	tail limpness
1.5	tail limpness and hind leg inhibition
2.0	partial hind leg paralysis
2.5	partial hind leg paralysis with dragging of at least one hind leg
3.0	complete paralysis of hind legs
3.5	complete hind leg paralysis and unable to right the body when placed on the side
4.0	complete hind leg and partial front leg paralysis
4.5	complete hind leg and partial front leg paralysis and mouse is not alert
5.0	spontaneously rolling in the cage or death

https://hookelabs.com/protocols/eaeAI_C57BL6.html

B. Luxol fast blue (LFB) staining * of demyelinated spinal cord with/without Eosin counterstaining

Slides of spinal cord paraffin section (5µm thick)

↓

Deparaffinize and hydrate

↓

Incubate with 0.1% LFB solution, overnight at 60°C

↓

Differentiate with 0.5% lithium carbonate solution, 1 min at room temperature

↓ | ↓ counter stain with Eosin Y solution, 1 min at room temperature ↓

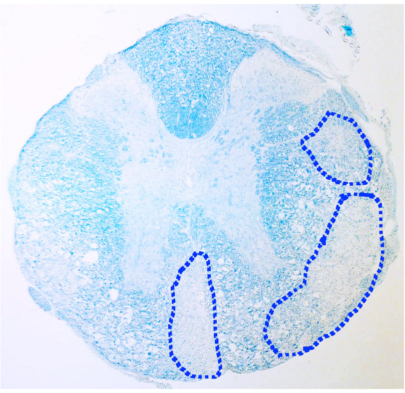

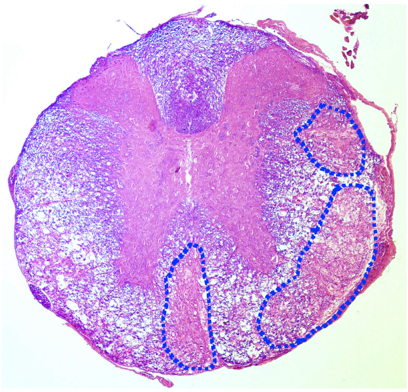

* Yoo IH, Kim MJ, Kim J, Sung JJ, Park ST, Ahn SW. The Anti-Inflammatory Effect of Sulforaphane in Mice with Experimental Autoimmune Encephalomyelitis. J Korean Med Sci 34, e197 (2019).
